# Caffeine inhibits gene conversion by displacing Rad51 from ssDNA

**DOI:** 10.1093/nar/gkv525

**Published:** 2015-05-27

**Authors:** Michael Tsabar, Jennifer M. Mason, Yuen-Ling Chan, Douglas K. Bishop, James E. Haber

**Affiliations:** 1 Department of Biology and Rosenstiel Basic Medical Sciences Research Center, Brandeis University, Waltham, MA 02454, USA; 2 Department of Radiation and Cellular Oncology and Department of Molecular Genetics and Cell Biology, University of Chicago, Chicago, IL 60637, USA

## Abstract

Efficient repair of chromosomal double-strand breaks (DSBs) by homologous recombination relies on the formation of a Rad51 recombinase filament that forms on single-stranded DNA (ssDNA) created at DSB ends. This filament facilitates the search for a homologous donor sequence and promotes strand invasion. Recently caffeine treatment has been shown to prevent gene targeting in mammalian cells by increasing non-productive Rad51 interactions between the DSB and random regions of the genome. Here we show that caffeine treatment prevents gene conversion in yeast, independently of its inhibition of the Mec1^ATR^/Tel1^ATM^-dependent DNA damage response or caffeine's inhibition of 5′ to 3′ resection of DSB ends. Caffeine treatment results in a dosage-dependent eviction of Rad51 from ssDNA. Gene conversion is impaired even at low concentrations of caffeine, where there is no discernible dismantling of the Rad51 filament. Loss of the Rad51 filament integrity is independent of Srs2's Rad51 filament dismantling activity or Rad51's ATPase activity and does not depend on non-specific Rad51 binding to undamaged double-stranded DNA. Caffeine treatment had similar effects on irradiated HeLa cells, promoting loss of previously assembled Rad51 foci. We conclude that caffeine treatment can disrupt gene conversion by disrupting Rad51 filaments.

## INTRODUCTION

Repair of DNA double-strand breaks (DSBs) is highly conserved within eukaryotic cells. Cells arrested in G1 predominantly repair DSBs by non-homologous end-joining (NHEJ). In this process, cells re-join the broken ends (1,2). After the cells pass ‘start’, on their way to initiate S phase, the main pathway of repair shifts to homologous recombination (HR) (2–4). Initially, Cdk1 activation facilitates the 5′ to 3′ resection of the broken ends, leaving 3′ single-stranded DNA (ssDNA) tails that are first coated by replication protein A (RPA). Rad52 is recruited to RPA-coated ssDNA and facilitates the formation of a filament of the Rad51 recombination protein (5–8). Two Rad51 paralogs, Rad55 and Rad57, assist in filament formation and stabilization (6,9–10). After the Rad51 filament forms, it facilitates a search for homology throughout the genome and promotes strand invasion between the ssDNA and homologous double-stranded DNA (dsDNA). Strand invasion is followed by the initiation of DNA synthesis from the 3′ end of the invading strand and eventual repair of the DSB (6,11). Repair can occur by gene conversion (GC) if the template sequence (a sister chromatid, homologous chromosome or an ectopic donor) contains homology to both sides of the DSB, or by break-induced replication (BIR) if only one end of the DSB is capable of pairing with homologous sequences (12–15). Recombination can also occur when the DSB is flanked by homologous sequences. In this process, termed single-strand annealing (SSA), resection exposes complementary strands of the two flanking homologies that can then anneal in a Rad52-dependent, but Rad51-independent manner, leading to a deletion.

Rad51 is a homolog of bacterial RecA that forms a right-handed helical filament on ssDNA or dsDNA. The adenosine triphosphatase (ATPase) catalytic domain encompasses the Walker A and Walker B motifs (12,14). Formation of the Rad51 filament requires binding to adenosine triphosphate (ATP) but not ATP hydrolysis (16). Rad51 filament disassembly has been shown to require ATP hydrolysis (17–19). In budding yeast, the Rad51 ATPase-defective Rad51-K191A mutant is unable to bind DNA, while Rad51-K191R loads on ssDNA, although with slower kinetics than the wild type (16,20–21). Importantly, although the rad51-K191R mutant is defective in repair, the defect can be rescued by blocking filament disassembly, indicating that the filaments formed by the mutant can function in homology search and strand exchange *in vivo* (22) a property supported by biochemical analysis and by studies of the analogous mutant in chicken DT40 cells (23).

Like RecA (24), Rad51 contains two DNA binding sites. The high affinity site (site I) binds ssDNA and forms the Rad51 filament, whereas the lower affinity site (site II) is used to associate the ssDNA-bound filament with the dsDNA sequences during the homology search (25,26). Mutation of three arginines to alanines in site II (denoted as Rad51-II3A) allows filament formation *in vitro* and Rad51 focus formation *in vivo*; however, Rad51-II3A bound to ssDNA does not facilitate joint molecule formation *in vitro* and exhibits sensitivity to ionizing radiation (IR) comparable to *rad51*Δ *in vivo* (25).

Several factors regulate the Rad51 filament. The Srs2 helicase is a DNA-dependent ATPase that displaces Rad51 from ssDNA and promotes recovery from the DNA damage checkpoint (27,28). The Swi2/Snf2-related translocases Rad54, Rdh54 and Uls1 play redundant roles in removing Rad51 non-specifically bound to dsDNA, thus releasing sufficient Rad51 to bind to ssDNA and promote HR (29–31). In *rdh54*Δ over-expression of Rad51 is toxic (29). Moreover, while Rad54 is not required to construct the Rad51 filament or to facilitate strand invasion, it is required in subsequent chromatin/recombinosome remodeling steps to initiate new DNA synthesis at the donor locus (6,31–32).

Caffeine treatment has been shown to inhibit HR in several ways. First, caffeine blocks activation of the PI3-like protein kinases ataxia telangiectasia mutated (ATM) and ATM and Rad3-related (ATR) that cause G2/M cell cycle arrest to allow cells sufficient time to repair DNA damage before mitosis is initiated (33–35). However, more recent work showed that caffeine failed to further radio-sensitize both mammalian and chicken DT-40 cells that are deficient for HR but have an intact checkpoint (36–38). These observations might indicate that the checkpoint kinases are essential for HR, but they could also indicate that caffeine treatment affects HR directly. Second, in an accompanying paper we show that caffeine impairs the 5′ to 3′ resection of DSB ends by inhibiting protein synthesis, leading to the consequent rapid proteasomal degradation of both Sae2 and Dna2, two key resection proteins. The impairment of resection, however, cannot alone account for inhibition of all HR as GC and BIR have been shown to be efficient in mutants that impair resection (39–42).

Recently caffeine was shown to inhibit gene targeting in mammalian cells independently of ATM and ATR inhibition and to interfere with the fidelity of joint molecule formation *in vitro* but not impair the ssDNA bound Rad51 filament itself (43). Here we demonstrate that caffeine prevents GC in yeast even when very little resection is required, by directly interfering with the integrity of the Rad51-ssDNA filament. We show that caffeine leads to eviction of yeast Rad51 from established ssDNA-bound filaments in a dose-dependent manner. Cytological analysis suggests that caffeine has a similar effect on Rad51 foci in mammalian cells. This work suggests a mechanism by which caffeine interferes with joint molecule formation and provides further insight into the manner by which caffeine prevents HR.

## MATERIALS AND METHODS

### Yeast strains and plasmids

All yeast strains used in this study are described in Table 1. In chromatin immunoprecipitation (ChIP) experiments JKM139 and JKM179 were used interchangeably. Deletions of open reading frames were carried out using single-step-polymerase chain reaction-mediated transformation of yeast colonies as described (44) or introduced by genetic crosses. Primer sequences are available on request. The Rad55-Rad57 2 μ plasmid was a gift from W.-D. Heyer (UC Davis, CA, USA), pRS315 wild-type (WT) *RAD51* was described in (45). pRS315 *rad51K191R* was constructed by using site-directed mutagenesis using QuikChange Lightning kit (Agilent Technologies).

**Table 1.** Strain list

Strain name	Genotype
JKM179	*hoΔ hml::ADE1 MATα hmr::ADE1 ade1-110 leu2,3-112 lys5 trp1::hisG ura3-52 ade3::GAL:HO*
JKM139	*JKM179 isogenic, MATa*
YML002	*JKM139 (HO cut site deleted) Cen3HOcs::HPH 2 kb homology to the left of the HOcs inserted to the right of Cen3, 97700-97800 Ch6::HOcs-NAT*
tGI354	*JKM139 MATa-inc (+CA), arg5,6::MATa-HPH*
MT03	*hoΔ HMLα MATα HMRα-BamHI::URA3 ade1 leu2 trp1::hisG ura3-52 ade3::GAL:HO*
YJL112	*MT03 tel1::TRP1 mec1::NAT sml1::KAN*
YFD0918	*JKM179 atg1::KAN*
YFD0247	*JKM139 srs2::LEU2*
AWY313	*JKM179 sgs1::KAN*
YSL305	*JKM139 rad55::LEU2*
MT101	*JKM139 ura3-52::TIR-LEU2 rad52-AID::KAN*
MT104	*JKM179 RAD52-FLAG::KAN*
MT109	*JKM179 pADH1-RAD51 LEU2 2μ*
MT113	*JKM179 pRAD55-RAD57 URA3 2μ*
MT121	*tGI354 pADH1-RAD51*
MT123	*JKM179 rad51::HPH pRAD51-LEU2*
MT124	*JKM179 rad51::HPH prad51K191R-LEU2*
MT127	*hoΔ HMLα MATa hmr::ADE1 ade1-110 leu2,3-112 lys5 trp1::hisG ura3-52 ade3::GAL:HO rad54::KAN rdh54::URA3 uls1::LEU2*
MT151	*JKM179 RAD51-II3A::TRP1*

### ChIP

ChIP was carried out as described previously (46). α-ScRad51 antibodies were a generous gift of A. Shinohara (University of Osaka, Osaka, Japan) and Douglas Bishop (University of Chicago, Chicago, IL, USA). α-ScRfa1 antibody was a gift of S. Brill (Rutgers, NJ, USA). Rabbit α-Rad52 was produced by Yuen-Ling Chan at the Bishop lab.

### Western blotting

Western blotting was carried out using the trichloroacetic acid protocol described by Pellicioli *et al*. (47). Samples were quantified using Quantity One software. Rad51 signal was normalized to loading control of the same time-point and to 0 h. α-ScRad51 antibodies were a generous gift of A. Shinohara. α-ScRho1 was a generous gift from Satoshi Yoshida (Brandeis, MA, USA).

### Southern blotting

Southern blot analysis was performed as previously described (40). Genomic DNA was purified and digested with EcoRI. Digested DNA was probed with a *MAT* distal probe (48). Donor signal served as loading control. Bands were normalized to the loading control and then to the 0 h.

### DNA binding assay

The effect of caffeine on the binding of yeast Rad51 or Rad52 to ssDNA was assayed by the fluorescence polarization method as described previously (49) with the following modifications. An 84-mer ssDNA conjugated with Alexa Fluor-488 at the 5′ (sequence: 5′GGTAGCGGTTGGGTGAGTGGTGGGGAGGGTCGGGAGGTGGCGTAGAAACATGATAGGAATGTGAATGAATGAAGTACAAGTAAA-3′; synthesized by Integrated DNA Technologies) was used at 200 nM nucleotides (2.4 nM molecule). The binding reactions were performed at 37°C for 30 min in buffer B (25 mM Tris-HCl (pH 7.8); 5 mM MgCl_2_, 3 mM ATP, 1 mM DTT, 20 mM NaCl_2_, 50 μM CaCl_2_ and 100 μg/ml bovine serum albumin (BSA)). The fluorescence polarization (in mP units) was measured using a Tecan Infinite F200 PRO plate reader. All binding conditions were performed in triplicate, and the mean values plotted with standard deviation. Caffeine had no effect on fluorescence polarization in the absence of added protein (data not shown). The methods used to purify the ScRad51 and ScRad52 protein were described previously (25).

### DNA intercalation assay

The method of Zelensky *et al*. (43) was adopted to examine the effect of intercalating agents on DNA. Briefly, negatively supercoiled *Escherichia coli* plasmid pBlueScript was nicked by DNase I, phenol:chloroform extracted, ethanol precipitated and dissolved in water as starting material for the subsequent assay. The nicked plasmid (320 ng) was first incubated at 37°C for 30 min in 20 μl of 1 × T4 DNA ligase buffer (NEB) containing different concentrations of caffeine, chloroquine or ethidium bromide. Second, 0.5 μl ligase (NEB) was added to the mixture which was incubated at room temperature for 30 min. The reaction was stopped by phenol:chloroform extraction, followed by another chloroform extraction to deproteinize and remove the intercalating agents. The resulting topoisomers in half of the reaction mixture (10 μl) were separated by electrophoresis in 1D agarose gel (1%) in 1 × TAE buffer at room temperature at 6 v/cm for 4 h. For the 2D gel electrophoresis, the remaining 10 μl reaction mixture was first fractionated in 1D gel as above, followed by rotating the gel 90° clockwise in 1 × TAE running buffer containing 5 μg/ml chloroquine. The 1D gel was then soaked for 2 h in the same chloroquine-containing buffer before electrophoresis at 1.6 v/cm for 15 h at room temperature.

### ATPase assay

To examine the effect of caffeine on ssDNA-dependent ScRad51 ATPase activity: on ice, a 10 μl reaction mixture containing buffer (20 mM HEPES, pH 7.5, 5 mM MgCl_2_, 1 mM DTT), 100 μM γ-^32^P-ATP, with or without caffeine (0, 10, 25, 40, 50 and 60 mM), ScRad51 (2.5 μM) and M13mp18 ssDNA (7 μM nucleotides or 1 nM) was assembled. ssDNA was added last to the mixture to initiate ATP hydrolysis. Incubation was at 37°C for 40 min. The reaction was stopped by adding 2 μl of 10 N formic acid. Two microliter of each sample was spotted on a thin layer plate (TLC PEI-Cellulose F, Merck) and chromatogram developed in solvent (0.5 M LiCl_2_ and 1 M formic acid). The TLC plate was dried and analyzed by phosphor-imaging to assess the level of ATP hydrolysis.

### D-loop assay

The assay was performed essentially as described previously (25) with the following modifications. *Saccharomyces cerevisiae* Rad51 (1.2 μM) was pre-incubated with Rad52 (2.4 μM) on ice for 20 min before adding to ssDNA (90 mer, 3.6 μM nucleotide or 40 nM).

### Mammalian cells culture conditions

HeLa cells were grown in Dulbecco's modified Eagle's medium (DMEM) (Invitrogen) supplemented with 10% fetal bovine serum, and 1X penicillin/streptomycin (Invitrogen). Cells were cultured at 37°C with 5% CO_2_.

### Immunofluorescence

HeLa cells (1.0 × 10^5^) were plated in 6-well dishes (Corning) containing a 22 × 22 glass coverslip (Fisherbrand) and allowed to adhere overnight. Cells were irradiated with 6 Gy using a Maxitron generator and allowed to recover for 4 h before the addition of 30 mM caffeine. Cells were fixed 1 h after treatment with caffeine. Cytosolic proteins were pre-extracted with HEPES/TritonX-100 buffer (20 mM HEPES pH 7.4, 0.5% Triton X-100, 50 mM NaCL, 3 mM MgCl_2_, 300 mM sucrose in ddH_2_O) at room temperature for 10 min. Cells were subsequently fixed with 3% para-formaledhyde, 3.4% sucrose in phosphate buffered saline (PBS) for 10 min at room temperature. Cells were blocked in 1% BSA in PBS for 15 min and stained with human RAD51 serum (kind gift from Akira Shinohara, Dilution 1:1500) and RPA antibodies (Calbiochem (NA18), Dilution 1:2000) overnight at 4°C. Cells were washed with PBS and stained with Alexa Fluor conjugated secondary antibodies (Invitrogen, Dilution 1:1000) for 1 h at room temperature. Cells were washed with PBS and allowed to air dry. Coverslips were mounted on slides with vectashield containing 4′,6-diamidino-2-phenylindole. To image cells, 1 μ sections were acquired on a scanning laser confocal microscope (LSM510, Zeiss) using LSM software. Images were acquired with a 60X objective. Z stacks were compressed into a single image and analyzed using ImageJ software (NIH). The numbers of RAD51 and RPA foci in at least 75 random nuclei from three independent experiments were counted. Statistical significance was determined using the Wilcoxon Rank Sum test.

## RESULTS

### Caffeine treatment prevents GC in budding yeast

**Figure 1.**
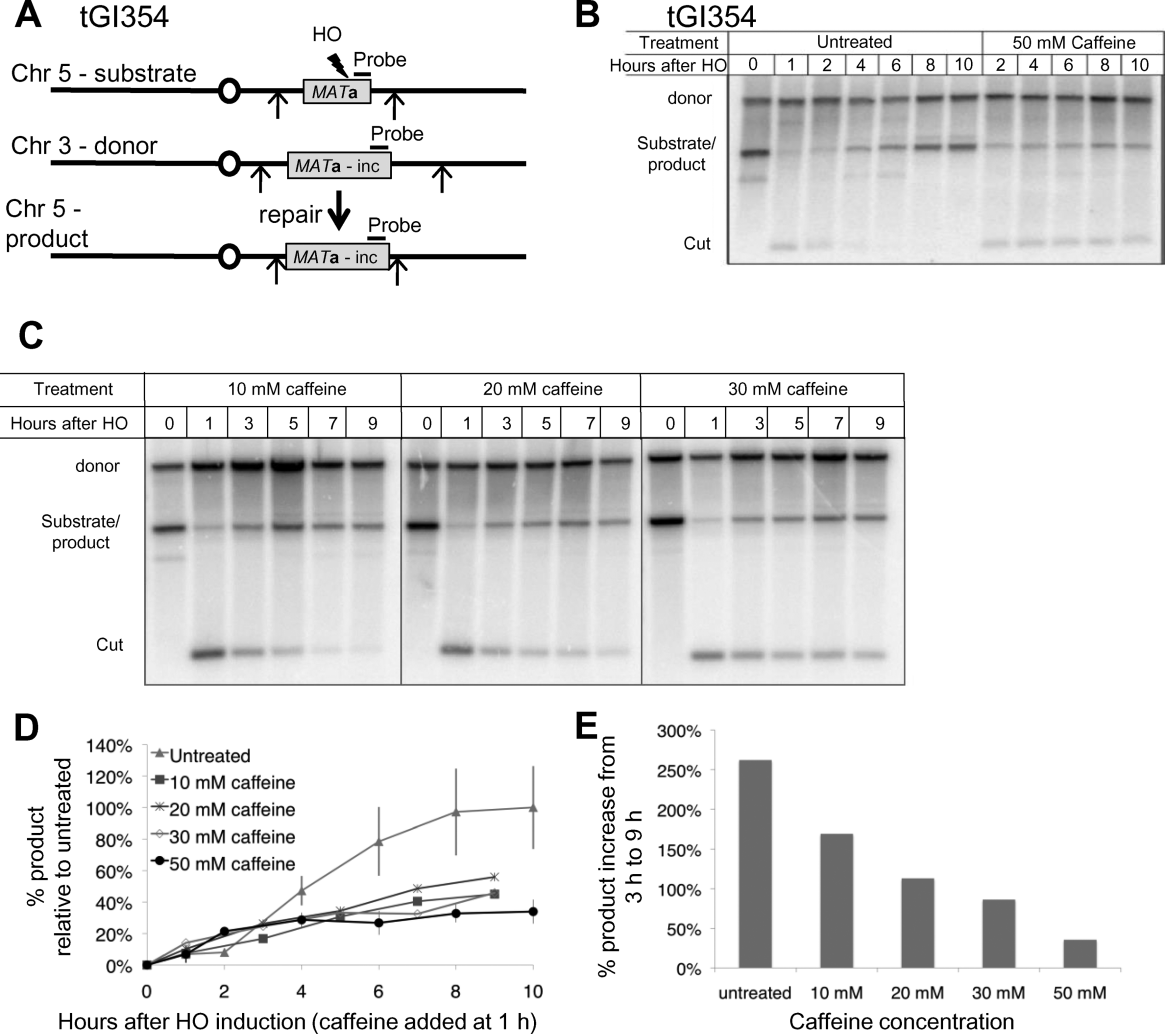
Caffeine treatment impairs GC. (**A**) Diagram of ectopic GC in tGI354. The *MAT*a-inc donor on Chr 3 cannot be cut by HO. Arrows indicate EcoRI restriction sites. (**B**) Repair in tGI354. Culture split 1 h after HO induction, half treated with 50 mM caffeine. In addition to a marked reduction in product, the HO-cut fragment is more stable because of a defect in resection. (**C**) Southern blots to assay repair by GC after 10–30 mM caffeine treatment. Caffeine was added 1 h after HO induction. (**D**) Quantification of repair in tGI354 treated with caffeine at different concentrations 1 h after HO induction. Product signal was normalized to the loading control (donor) and the untreated maximal repair signal (10 h). Untreated *n* = 3, 10–30 mM caffeine *n* = 1, 50 mM caffeine *n* = 3. (**E**) Percent increase in GC product formation from 3 to 9 h with different caffeine concentrations. Product at 9 h after HO induction (8 h after caffeine treatment) was divided by the product 3 h after HO induction (2 h after caffeine treatment).

Caffeine treatment slows resection by leading to the proteasomal degradation of Dna2 and Sae2 (50). However, previous studies have shown that reduced resection *per se* does not prevent GC when only a few hundred bp of DNA need to be resected (41,51–52). Specifically, depletion of Sae2 in a strain lacking *SGS1*, the RecQ helicase that interacts with Dna2 to promote its resection activity (51,52), did not inhibit GC (41), despite reducing resection. Nevertheless, caffeine treatment inhibits interchromosomal GC where the donor is nearly identical to the region surrounding the DSB (Figure 1A). A *MAT***a** locus introduced in Chr 5 can be cleaved by a galactose-inducible HO endonuclease and repaired using a *MAT***a**-inc donor on Chr 3 which contains a single bp substitution that prevents HO cleavage (53). In this strain only a few hundred bp of resection are required, as the DSB has near-perfect homology to the donor. Caffeine was added at final concentrations of 10, 20, 30 and 50 mM, 1 h after inducing HO expression. Surprisingly, caffeine treatment severely inhibited GC at all concentrations tested (Figure 1C and D), with higher concentrations resulting in a more severe defect (Figure 1D and E). In the untreated samples there was a 260% increase in product signal from 3 to 9 h after HO induction. In the 10 mM caffeine-treated samples the product rose by 170% at the same time frame, whereas in the samples treated with 50 mM caffeine there was only a 36% increase in product from 3 to 9 h after HO induction (Figure 1E). These results indicate that the inhibition of HR is not limited to impairment of resection. The effect of caffeine on the resection-dependent loss of the HO endonuclease-cut region is evident in Figure 1C.

### Caffeine treatment prevents GC independently of Mec1 and Tel1 inhibition

**Figure 2.**
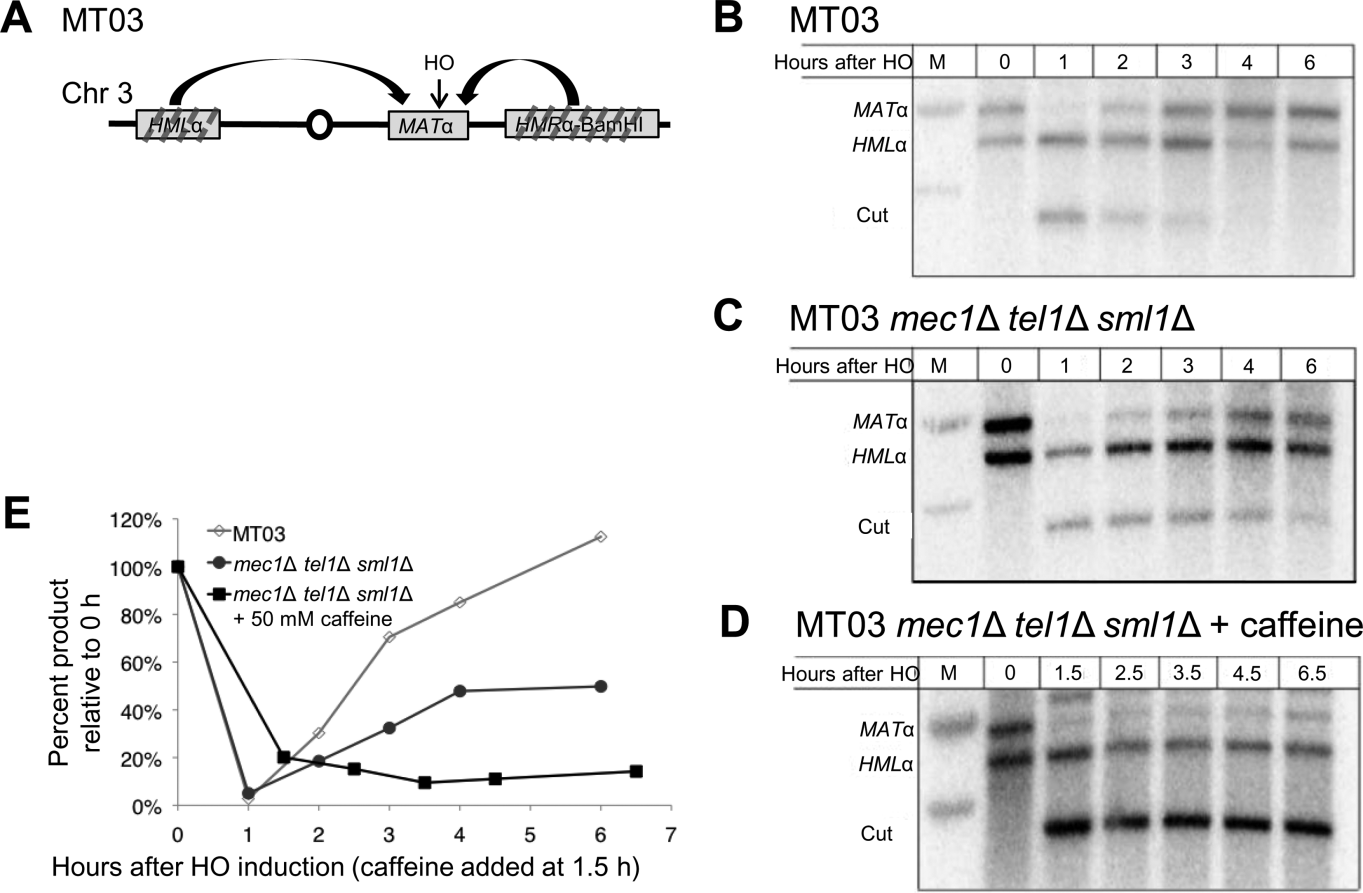
Caffeine inhibition of GC is independent of the DNA damage checkpoint. (**A**) Diagram of intrachromosomal gene conversion strain MT03. (**B**) Repair in MT03. HO was induced by galactose for 1 h, after which expression was shut off by addition of dextrose. (**C**) Repair in MT03 *mec1*Δ *tel1*Δ *sml1*Δ. Culture was treated as in (B). (**D**) Repair in caffeine treated MT03 *mec1*Δ *tel1*Δ *sml1*Δ. Culture was treated as in (B). 50 mM caffeine added 0.5 h after dextrose. (**E**) Quantification of repair in MT03 derivatives. Signal normalized to the 0 h signal.

Caffeine is commonly used as an inhibitor of the checkpoint kinases ATM and ATR (Tel1 and Mec1 in yeast) (27,54–59). To see if caffeine treatment prevents GC by inactivation of Mec1 and Tel1, we used a strain in which a DSB at *MAT*α can be repaired using one of the two homologous donors, *HML*α or *HMR*α-BamHI (Figure 2A) (60). Here again there is only a minimal requirement for resection as the donors are almost completely homologous to both sides of the DSB. Because repair results in a product that can also be cut by HO, its expression was shut off by adding dextrose 1 h after HO induction (60). In the accompanying paper (50) we show that under these conditions 10% of the product can be attributed to NHEJ. In the WT cells, repair was almost complete in 5 h (Figure 2B and E). The triple deletion, *mec1*Δ *tel1*Δ *sml1*Δ (deletion of *SML1* allows viability in the absence of *MEC1* in budding yeast), reduced GC to 50% of WT (Figure 2C and E). This decrease in repair efficiency could be attributed to the absence of a DNA damage checkpoint that modifies several recombination proteins (61–64). However, adding 50 mM caffeine 30 min after dextrose addition severely inhibited repair (Figure 2D and E). This result demonstrates that caffeine prevents GC in a manner distinct from Mec1 and Tel1 inhibition.

### Rad51 is evicted from ssDNA in response to caffeine treatment

**Figure 3.**
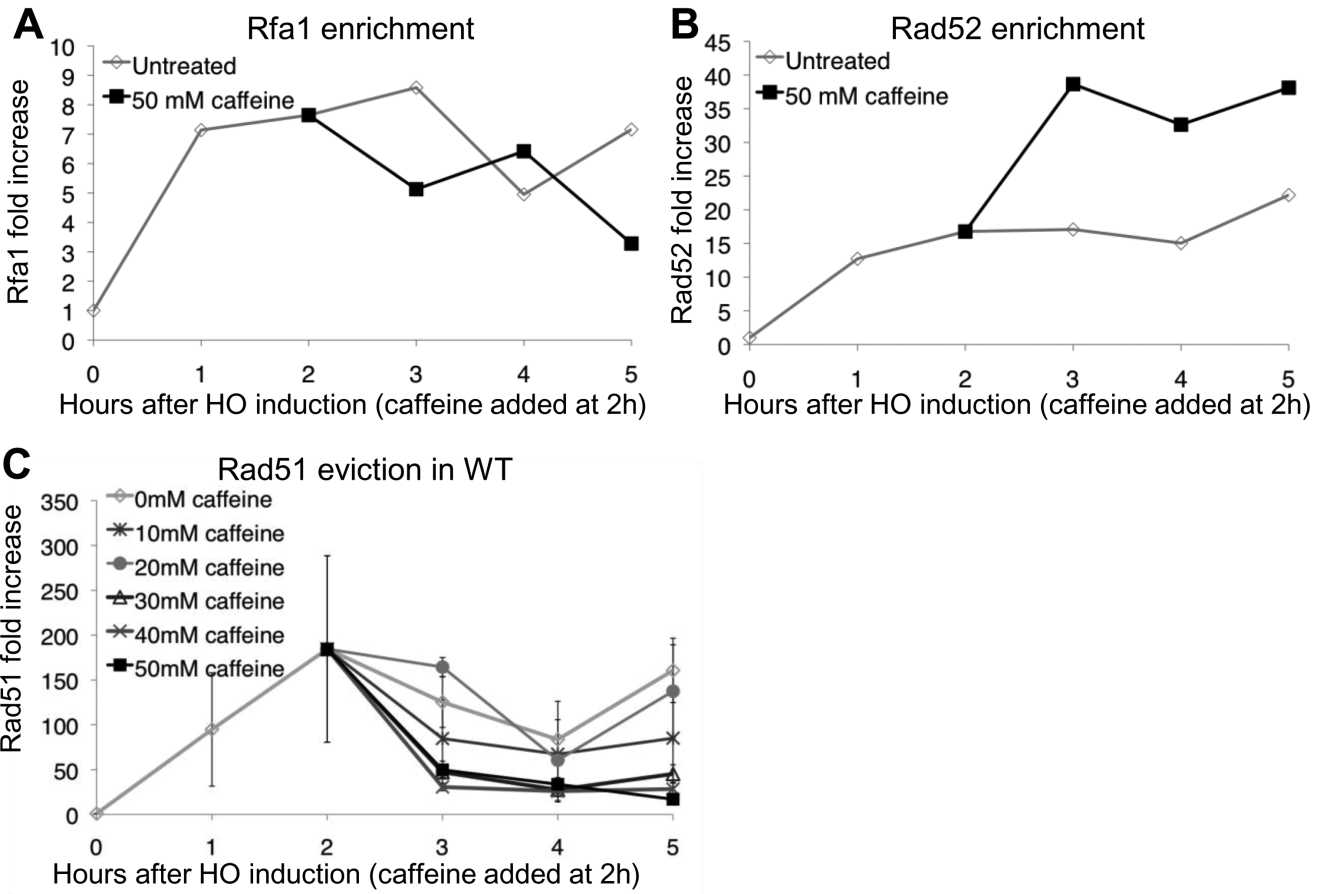
Caffeine treatment leads to Rad51 eviction. (**A**) Rfa1 ChIP 100 bp proximal to the DSB at *MAT* in a donorless strain. ChIP signal measured 100 bp distal to the DSB. Untreated samples are shown in gray, 50 mM caffeine was added 2 h after DSB induction (black). (**B**) Rad52 ChIP 100 bp proximal to the DSB at *MAT* in a donorless strain. ChIP signal measured 100 bp distal to the DSB. Untreated samples are shown in gray, 50 mM caffeine was added 2 h after DSB induction (black). (**C**) Rad51 ChIP 100 bp proximal to the DSB at *MAT* in a donorless strain. Increasing concentrations (10–50 mM in 10 mM increments) were added 2 h after DSB induction. Error bars represent ranges.

GC could be inhibited if both strands of the region adjacent to the DSB that shares homology with an ectopic donor were degraded after caffeine treatment. In this and other experiments described below we used a *MAT*α *hml*Δ *hmr*Δ strain in which a DSB cannot be repaired in >99% of the cells (65). To test if the ssDNA around the DSB is intact we used ChIP to assess if the ssDNA remains bound by RPA after caffeine treatment (Figure 3A and Supplementary Figure S1A). Using an antibody against the largest subunit of RPA, Rfa1, we found that, 100 bp from the DSB, Rfa1 levels increased 8-fold 1 h after HO induction. Caffeine treatment 2 h after HO induction did not significantly alter Rfa1 binding to ssDNA either 100 bp from the DSB (Figure 3A) or 5 kb from the break (Supplementary Figure S1A). These observations suggest that RPA recruitment is not impaired by caffeine treatment and also demonstrates that caffeine treatment leaves intact the ssDNA that serves as substrate for RPA and Rad51.

Rad52 is recruited to RPA-bound ssDNA and is essential for HR in budding yeast (6,11–12,66–69). We asked if treatment with 50 mM caffeine affects Rad52 recruitment to the DSB. Rad52 signal 100 bp from the DSB reached a maximum of 15 fold enrichment 2 h after HO induction (Figure 3B). Interestingly, treatment with 50 mM caffeine 2 h after HO induction led to a further increase in the Rad52 signal as compared to the untreated signal (Figure 3B). Five kilobase from the DSB Rad52 ChIP signal increased and reached a maximum 3 h after HO induction and remained at a maximal level throughout the experiment (Supplementary Figure S1B). Treatment with 50 mM caffeine 2 h after HO induction did not affect Rad52 recruitment as well (Supplementary Figure S1B).

GC requires Rad51 filament formation to facilitate homology searching. In contrast to RPA and Rad52, treatment with 50 mM caffeine dramatically affected Rad51 binding (Figure 3C). Without caffeine, Rad51 levels, monitored by ChIP 100 bp from the irreparable DSB, were elevated 170-fold after 2 h relative to the level prior to DSB induction. Surprisingly, addition of 50 mM caffeine 2 h after formation of a DSB resulted in a nearly complete loss of Rad51 from the ssDNA within 1 h.

Because caffeine inhibited GC at concentrations as low as 10 mM we asked if Rad51 is displaced after treatment with lower caffeine concentrations as well. The eviction of Rad51 by caffeine could also be observed when the cells were treated with 40 and 30 mM caffeine 2 h after DSB induction. Treatment with 10 and 20 mM of caffeine prevented the further accumulation of Rad51 after treatment, but not the loss of the Rad51 signal (Figure 3C). These observations give rise to the possibility that caffeine treatment results in Rad51 eviction from the ssDNA at concentrations of 30 mM or greater, while at concentrations lower than 30 mM caffeine targets another essential step in GC such as joint molecule formation (43).

**Figure 4.**
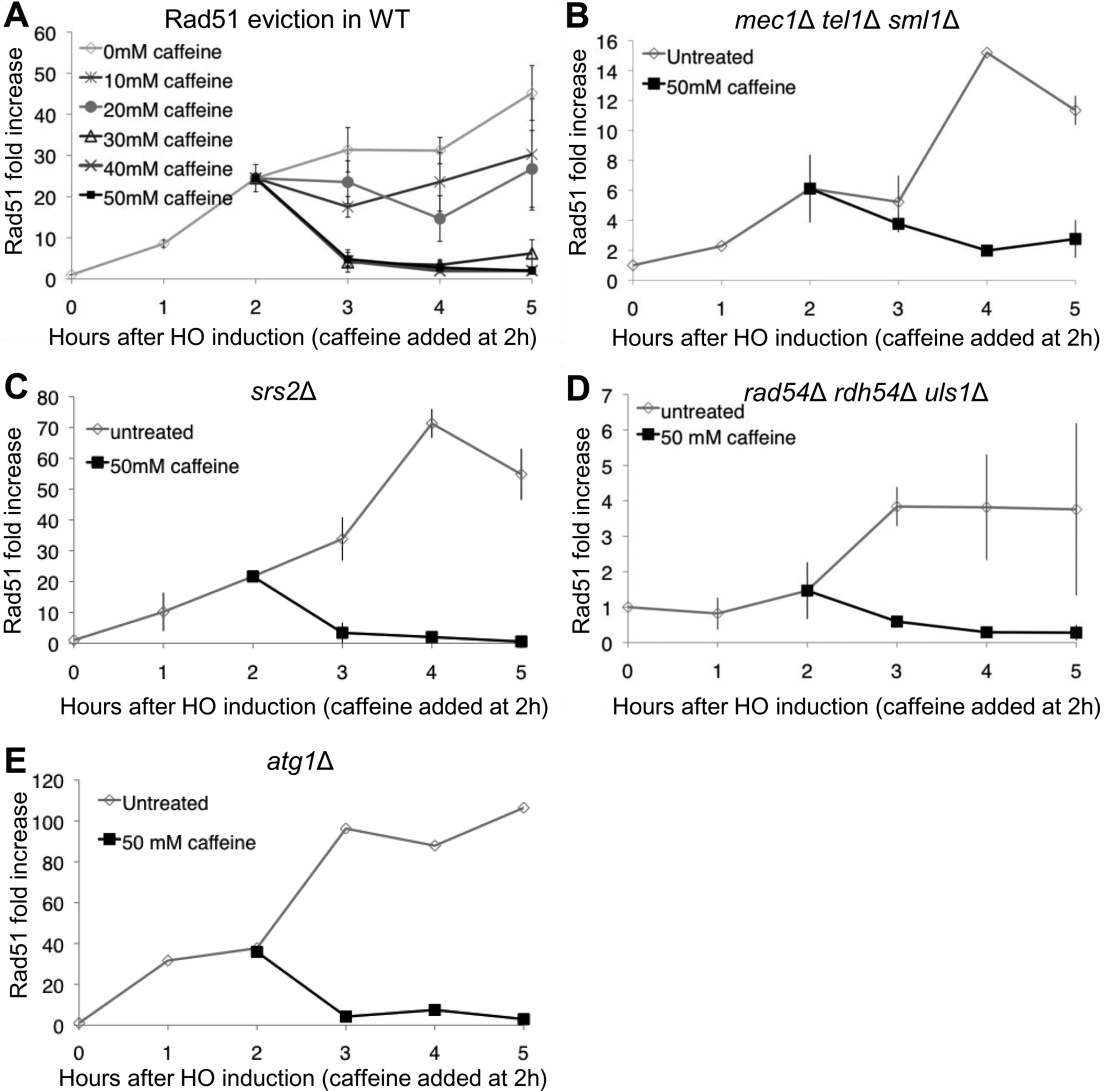
Caffeine treatment leads to Rad51 eviction independently of factors known to displace Rad51. (**A**) Rad51 ChIP 5 kb proximal to the DSB at *MAT* in a donorless strain. Increasing concentrations (10–50 mM in 10 mM increments) were added 2 h after DSB induction. Untreated *n* = 13, 10–40 mM caffeine *n* = 3, 50 mM caffeine *n* = 5. (**B**) Rad51 ChIP 5 kb proximal to the DSB at *MAT* in a *mec1*Δ *tel1*Δ *sml1*Δ donorless strain. (**C**) Rad51 loading in an *srs2*Δ strain. 50 mM caffeine added 2 h after HO induction. ChIP signals measured 5 kb from the DSB. Error bars represent ranges. (**D**) Rad51 loading in a *rad54*Δ *rdh54*Δ *uls1*Δ strain. 50 mM caffeine added 2 h after HO induction. ChIP signals measured 5 kb from the DSB. Error bars represent ranges. (**E**) Rad51 loading in an *atg1*Δ strain. 50 mM caffeine added 2 h after HO induction. ChIP signals measured 5 kb from the DSB.

Rad51 levels at a distance of 100 bp from the DSB plateau 2 h after HO induction (Figure 3C). To test the effect of caffeine treatment on the filament integrity further from the DSB end we monitored Rad51 enrichment 5 kb from the DSB (Figure 4A). At this distance Rad51 binding continues to increase throughout the time course and reaches 60-fold 5 h after HO induction. Similar to the observation at 100 bp, caffeine at concentrations of 30 mM or greater led to the complete loss of Rad51 from the resected DNA.

Loading of Rad51 was impaired in a strain lacking Mec1 and Tel1. Rad51 binding as measured by ChIP in a *mec1*Δ *tel1*Δ *sml1*Δ was delayed compared to WT and only reached 12-fold increase at 5 h. However, caffeine treatment at 2 h led to the loss of the Rad51 ChIP signal (Figure 4B) indicating that the eviction of Rad51 following caffeine treatment is independent of the checkpoint inhibition by caffeine.

It is possible that caffeine leads to Rad51 eviction by stimulating Rad51 to hydrolyze ATP, analogous to results in other systems (70,71). We therefore used a *rad51*Δ strain complemented by a centromere-containing plasmid with *rad51-K191R* expressed from its endogenous promoter to test for the involvement of ATP hydrolysis in dissociation of Rad51 filaments (Supplementary Figure S2A and B). *In vitro*, Rad51-K191R is defective in filament formation but after the filament formed it displayed greater stability than WT Rad51 (17). Our ChIP data confirmed that Rad51-K191R forms a filament slower than WT Rad51 (Supplementary Figure S2B). After 5 h, the Rad51-K191R ChIP signal increased 5-fold, and only reached 30-fold 8 h after HO induction. We treated the cells with 50 mM caffeine 5 h after HO induction to allow for sufficient Rad51 to load on the ssDNA. After caffeine treatment, the Rad51-K191R protein was rapidly evicted from the ssDNA (Supplementary Figure S2B), suggesting that Rad51's eviction is independent of ATP hydrolysis.

The loss of Rad51 from the ssDNA could be a result of dissociation of Rad51 from the filament in the presence of caffeine or by increasing non-specific interactions between the Rad51-bound ssDNA and non-homologous targets throughout the genome, as suggested by Zelensky *et al*. (43). We therefore asked if Rad51-II3A, which forms a filament but cannot facilitate binding to dsDNA in site II to form D-loops, is also sensitive to caffeine. Rad51-II3A associates with ssDNA as seen by ChIP, albeit not as efficiently as WT Rad51 (Supplementary Figure S2C). Treatment with 50 mM caffeine 2 h after HO induction resulted in the loss of Rad51-II3A from the ssDNA. This result suggests that the eviction of Rad51 is not a result of promiscuous interactions of Rad51 release after binding at non-homologous sites nor does eviction require the ability of Rad51 to form D-loops.

### Caffeine evicts Rad51 independent of the factors known to displace Rad51 from ssDNA and dsDNA

Caffeine may evict Rad51 by promoting Srs2 activity, which has been previously shown to dismantle the Rad51 filament (28). To test this possibility we performed Rad51 ChIP in an *srs2*Δ strain (Figure 4C). As previously shown, more Rad51 assembled around the DSB in an *srs2*Δ strain than in WT (72). However, after caffeine treatment, Rad51 was displaced. Therefore caffeine does not act through stimulating Srs2.

Rad54, Rdh54 and Uls1 play redundant roles in stripping Rad51 from non-specific association with dsDNA and thus increasing the pool of Rad51 that can from a filament on ssDNA (29–31). We tested if caffeine treatment leads to aberrant regulation of these translocases. The Rad51 ChIP signal in a *rad54*Δ *rdh54*Δ *uls1*Δ triple mutant is lower at 2 h after HO induction than in WT cells, consistent with the idea that, without these proteins, there is less Rad51 available to load on the resected ssDNA and/or that there is a higher general non-specific background signal before HO induction (Figure 4B). We cannot exclude that Rad51 loading may be impaired in the absence of these translocases. However, as in the *srs2*Δ strain, caffeine treatment in the *rad54*Δ *rdh54*Δ *uls1*Δ strain also leads to rapid loss of Rad51 signal. This observation demonstrates that caffeine acts independently from these three translocases.

### Rad51 is evicted independently of autophagy

In an accompanying paper (50) we show that caffeine treatment induces autophagy. To test if caffeine treatment leads to Rad51 eviction by inducing Rad51 degradation by autophagy we assayed Rad51 recruitment in an *atg1*Δ strain, which prevents autophagy (Figure 4E). Deleting Atg1 did not impair Rad51 recruitment to the resected ssDNA. Caffeine treatment led to the displacement of Rad51 from the ssDNA in *atg1*Δ cells as in WT. These data indicate that caffeine treatment affects the Rad51 filament independently of autophagy.

### Rad51 eviction after caffeine treatment is independent of the machinery that promotes Rad51 filament formation

Recent work has shown that Rad52 undergoes post-translational modifications such as sumoylation (73,74). Sumoylated Rad52 rescues the DNA damage sensitivity of *srs2*Δ, possibly by counteracting the excessive Rad51 activity that occurs in the absence of Srs2 (72). Therefore it is possible that caffeine treatment leads to Rad51 eviction through regulating Rad52 post-translational modifications that might render Rad52 inactive thus leading to loss of the Rad51 filament. To test this hypothesis we constructed a Rad52 fused with an auxin inducible degron (Rad52-AID) (75). In the absence of auxin, Rad52-AID was able to mediate Rad51 filament formation, as seen by ChIP, although the Rad51 recruitment at 5 h after HO induction was lower than seen in WT Rad52 (Supplementary Figure S3A). When the culture was treated with 500 μM indole-3 acetic acid (IAA) 1 h prior to HO induction, Rad51 failed almost completely to bind the ssDNA, demonstrating that the addition of IAA degrades or inactivates Rad52-AID as fast as 1 h after exposure. When IAA was introduced 2 h after HO induction, Rad51 levels were initially unaffected. Addition of 50 mM caffeine 1 h after adding IAA led to the eviction of the filament as seen in WT cells, indicating that caffeine does not act on Rad51 through alterations of Rad52. We note that Rad51 was eventually lost 3 h after IAA treatment. This result may indicate that Rad52 is required to maintain the Rad51 filament after longer periods of time.

Rad51 is proposed to form a co-filament with the Rad55-Rad57 heterodimer, which is more resistant to the translocation activity of the anti-recombinase helicase Srs2 (10). In the absence of Rad55, Rad51 can bind to ssDNA, but its recruitment to the DNA is slower than WT and it is unable to promote stand invasion, a defect that can be overcome by over-expressing Rad51 (6,76–78). These observations raised the possibility that caffeine might cause Rad51 filament dissolution by inhibiting or displacing Rad55-Rad57. If caffeine acts by displacing Rad55, the filament formed in the absence of the heterodimer might be resistant to the treatment. Because Rad51 assembles on resected ssDNA in *rad55*Δ with slower kinetics than WT we allowed for a Rad51 filament to form for 6 h after HO induction before treating them with 50 mM caffeine (Supplementary Figure S3B). Caffeine treatment resulted in the eviction of Rad51 from the ssDNA. In addition, over-expression of both Rad55-Rad57 from a 2μ plasmid also failed to rescue the effect of caffeine on the Rad51 filament (Supplementary Figure S3C). These results suggest that the effect of caffeine is specific to Rad51, and is not mediated by the eviction or inhibition of Rad55-Rad57.

Because caffeine inhibits the DNA damage checkpoint, cells can escape the G2/M arrest brought about by this checkpoint and resume cell cycle progression. Caffeine treatment, therefore, could lead to Rad51 eviction by allowing the cells to resume the cell cycle. We tested if caffeine leads to Rad51 eviction in *S. cerevisiae* cells that are arrested in G2/M by nocodazole prior to HO induction (Supplementary Figure S3D). In the absence of caffeine, Rad51 loading in cells arrested by nocodazole treatment was the same as in untreated cells. Treatment with 50 mM caffeine 2 h after HO induction, however, led to Rad51 eviction in arrested cells, suggesting that caffeine treatment leads to Rad51 eviction independently of the cell cycle progression after treatment.

### Protein synthesis is required for maximal Rad51 accumulation on ssDNA

**Figure 5.**
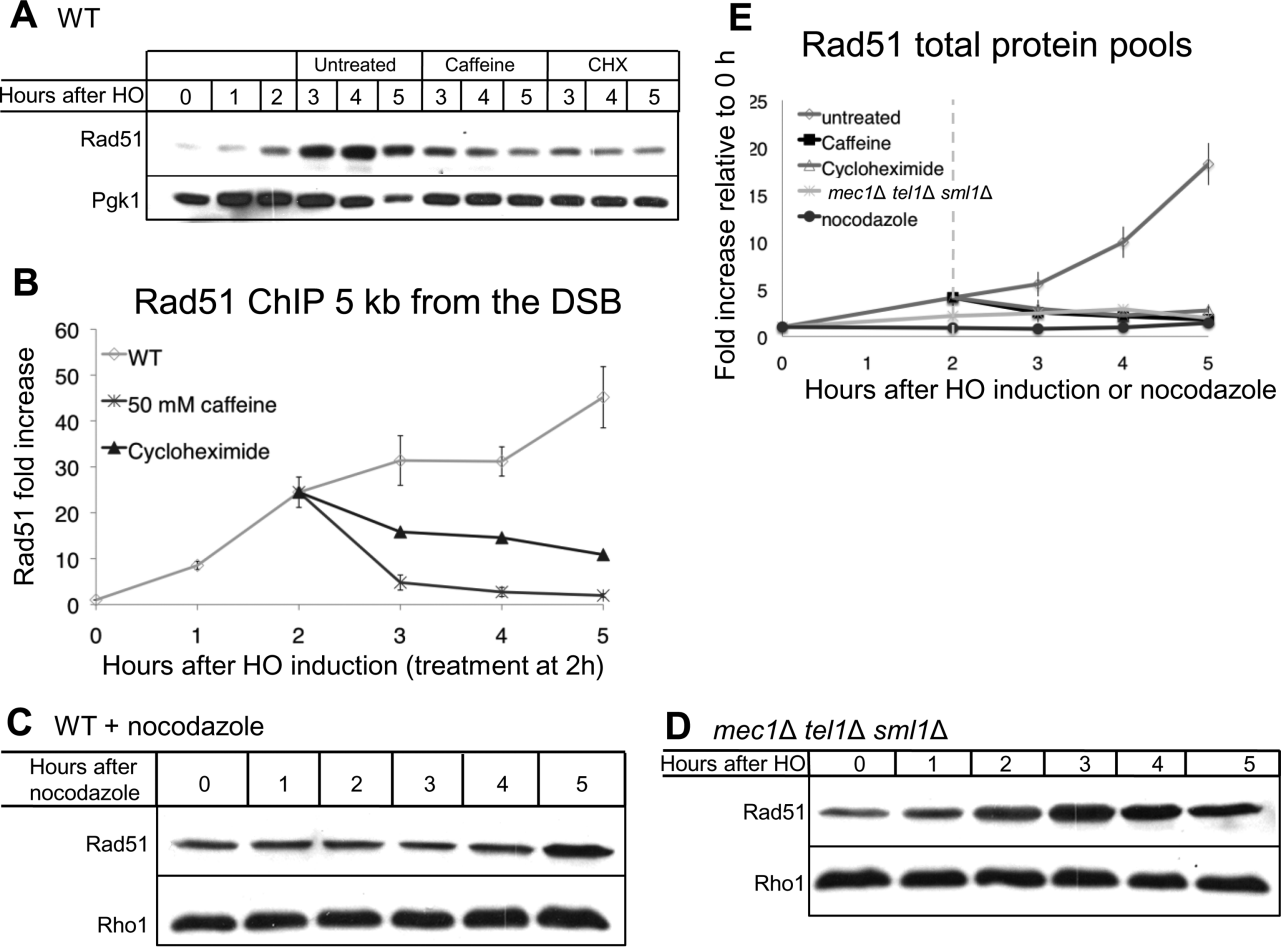
Rad51 is upregulated following a DSB in a DNA damage checkpoint-dependent manner. (**A**) Rad51 western blot in JKM179. 50 mM caffeine or 100 μg/ml CHX added 2 h after HO induction. (**B**) Rad51 ChIP 5 kb proximal to the DSB at *MAT* in a donorless strain. Untreated (diamonds), and either 50 mM caffeine (squares) or 100 μg/ml CHX (triangles) added 2 h after HO induction. Error bars indicate standard error of the mean. Untreated *n* = 13, rapamycin *n* = 2, CHX *n* = 1, 50 mM caffeine *n* = 5. (**C**) Rad51 western blot after 15 μg/ml nocodazole addition. Nocodazole added at 0 h. Three hours after nocodazole treatment >95% of the cells were arrested in dumbbell state. (**D**) Rad51 western blot after induction of a DSB in a *mec1*Δ *tel1*Δ *sml1*Δ strain. (**E**) Quantification of western blots shown in (C–E). Rad51 protein levels were normalized to loading control (Rho1 or Pgk1) and to 0 h levels. Error bars represent standard deviations for untreated, and ranges for caffeine and CHX treatments and for *mec1*Δ *tel1*Δ *sml1*Δ.

Caffeine treatment inhibits proteins synthesis in yeast and other organisms (79), so it was possible that one explanation for the loss of Rad51 ChIP signal is that caffeine prevents an induction of Rad51 protein levels. In yeast, X-ray irradiation leads to upregulation of Rad51 transcription in a cell cycle-independent manner (80). Here, we demonstrated that the abundance of Rad51 monitored by a western blot is upregulated after creation of a single DSB (Figure 5A). Rad51 was present in the cell prior to DSB induction, but its steady state level increased ∼15-fold following DSB induction (Figure 5A and E). Treatment of the cells with 100 μg/ml cycloheximide (CHX) 2 h after DSB induction prevented the increase in protein abundance (Figure 5A and E). Similarly, 50 mM caffeine added 2 h after DSB induction prevented further accumulation of Rad51.

We then tested the effect of inhibiting protein synthesis on Rad51 filament formation by ChIP. Unlike caffeine treatment, CHX treatment did not lead to complete Rad51 eviction from the ssDNA as measured by ChIP (Figure 5B). In CHX-treated cells the Rad51 signal remained 10-fold higher 5 h after HO induction as compared to 0 h. We conclude that new protein synthesis is required for full Rad51 accumulation around a DSB. However, the observation that CHX treatments result in the prevention of further accumulation, but not in full eviction of Rad51 from ssDNA, suggests that Rad51 filament eviction from ssDNA after caffeine treatment is not fully explained by inhibition of protein synthesis.

Failure to repair a DSB leads to cell cycle arrest in G2/M. Rad51 protein synthesis could, therefore, be regulated either by the DNA damage or by arrest of the cell cycle. Unlike DSB induction, nocodazole treatment did not lead to accumulation of Rad51 (Figure 5C and E), indicating that the increase in Rad51 levels is not due to G2/M arrest. We then tested if Rad51 enrichment depends on the DNA damage checkpoint by inducing DSBs in *mec1*Δ *tel1*Δ *sml1*Δ cells. We found that, in the absence of the DNA damage checkpoint kinases, Rad51 levels increased only modestly (3-fold as compared to 15-fold in WT) in response to DNA damage (Figure 5D and E). The lower levels of Rad51 in the *mec1*Δ *tel1*Δ *sml1*Δ strain may contribute to the lower level of Rad51 filament formation (Figure 4B) and repair by GC (Figure 2C and E). Taken together, these results suggest that Rad51 accumulation after DNA damage depends to a large extent on the checkpoint kinases, but not on G2/M arrest.

The observation that caffeine treatment at 2h prevented the DNA damage-dependent accumulation of Rad51 could imply that caffeine disrupts filament formation by preventing sufficient accumulation of Rad51 protein levels. We therefore tested the effect of 50 mM caffeine treatment 4 h after HO induction. By that time Rad51 protein levels have already increased 10-fold compared to 0 h (Figure 5E). Following caffeine addition at 4 h, the Rad51 ChIP signal was lost (Supplementary Figure S4A); however, protein levels did not decrease to an observable extent (Supplementary Figure S4B). We conclude that caffeine impairs Rad51 filaments not by reducing Rad51 levels but by promoting the disassociation of Rad51 from the filament.

### Caffeine inhibits Rad51's ssDNA binding and homologous strand assimilation activity *in vitro*

**Figure 6.**
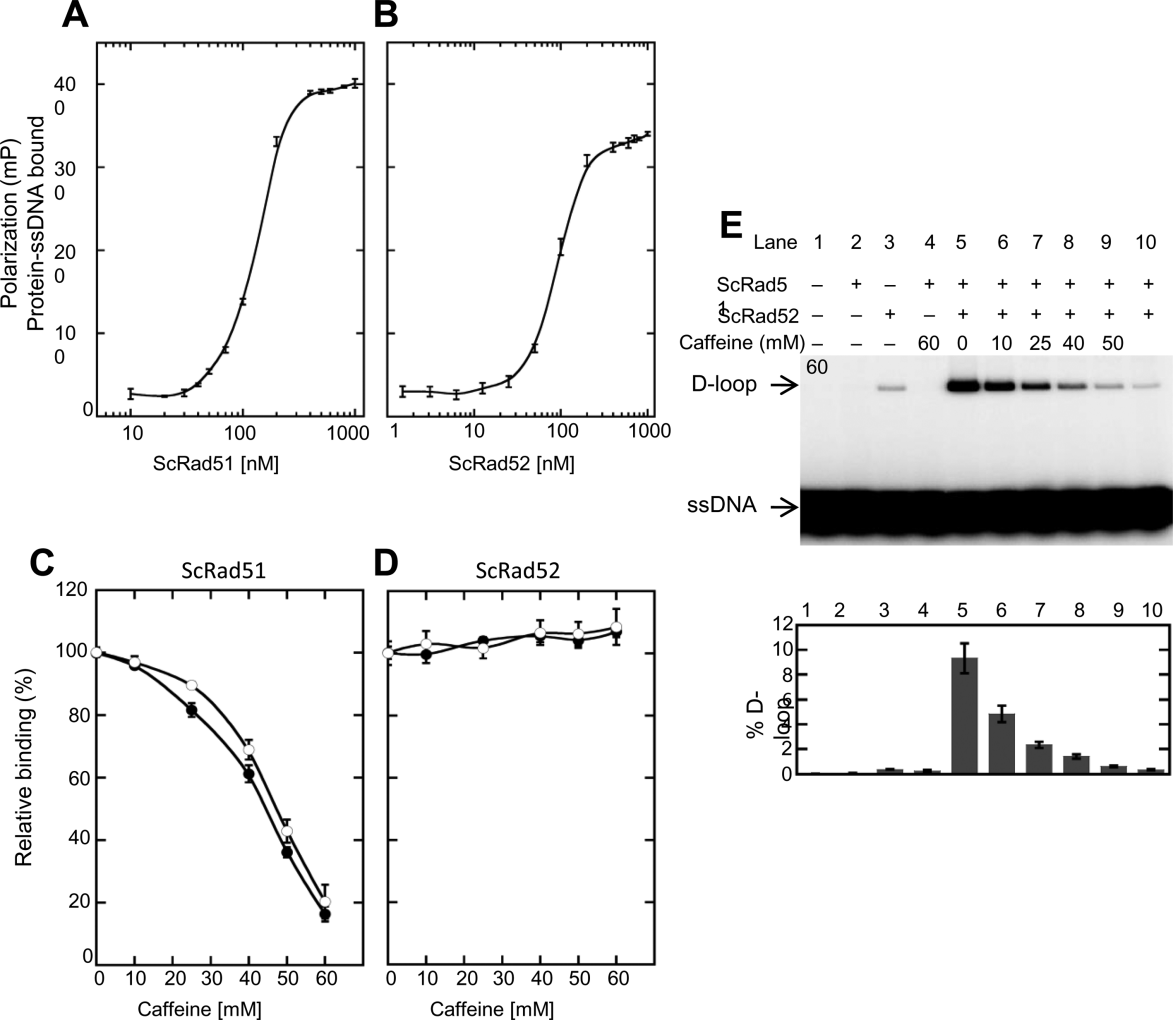
Effect of caffeine on ssDNA binding and D-loop activity. (**A**) Protein–DNA binding curves for Rad51 determined by fluorescence polarization assay. Protein at various concentrations was incubated with fluorescein-tagged 84-mer ssDNA (200 nM nucleotides or 2.4 nM molecules) in buffer B and incubated at 37°C for 30 min. Error bars represent standard deviation from triplicate trials. (**B**) Same as (A) except Rad52 replaced Rad51. (**C**) Caffeine (10, 25, 40, 50 and 60 mM) was added to pre-formed Rad51-ssDNA filaments (closed circles) or to Rad51 and ssDNA before filament formation (open circles). Rad51 was 150 nM. Binding was determined by fluorescence polarization as before. Data were normalized to the signal level observed in the absence of caffeine. (**D**) Same as in (C) except 100 nM Rad52 replaced Rad51. (**E**) The D-loop activity of Rad51 (1.2 μM) mediated by Rad52 (2.4 μM) was measured in the presence of increasing concentrations of caffeine. Upper panel: autoradiogram following electrophoretic separation of D-loops from free ^32^P-labeled ssDNA oligo substrate. Lower panel: quantitation of the autoradiogram shown in the upper panel together with results from two additional experiments (corresponding gels are not presented). Error bars represent standard deviation. Note: Rad52 has a low level of intrinsic D-loop activity which is not stimulated by Rad51 (25), as is evident in the control reaction shown in lane 3.

The results described above raise the possibility that caffeine acts directly to reduce the binding affinity of Rad51 for ssDNA. To test this possibility we measured the effect of caffeine on binding of Rad51 *in vitro* to an 84-nucleotide ssDNA substrate using a fluorescence polarization assay (81). First, the concentration of Rad51 required to obtain half-maximal binding to the ssDNA substrate was determined to be ∼150 nM (Figure 6A). This concentration was then used to assess the sensitivity of binding to caffeine. Caffeine was found to inhibit binding with 50% inhibition at 45 mM and 85% inhibition at 60 mM caffeine (Figure 6C). The degree of inhibition did not depend on whether or not Rad51 was allowed to bind DNA before addition of caffeine. These results indicate that caffeine can reduce the affinity of Rad51 for ssDNA in the same concentration range that blocks ssDNA binding of Rad51 following HO cleavage *in vivo*.

We also measured binding of purified Rad52 protein using the same ssDNA substrate. The concentration of Rad52 required for half-maximal binding was found to be ∼100 nM (Figure 6B). Caffeine showed no inhibition of Rad52 binding at 100 nM Rad52 for concentrations of caffeine of up to 60 mM (Figure 6D). Thus, caffeine inhibits Rad51-ssDNA binding under conditions that do not inhibit Rad52-ssDNA binding.

Caffeine was previously reported to inhibit the ability of human RAD51 to promote homologous strand assimilation of an ssDNA oligonucleotide into a supercoiled plasmid substrate (43). We used a similar ‘D-loop’ assay to test the ability of caffeine to inhibit *S. cerevisiae* Rad51's strand assimilation activity or D-loop activity. Rad51 requires addition of either Rad52 or Rad54 protein for this activity (67). We therefore asked if caffeine would inhibit Rad51's D-loop activity mediated by Rad52 (Figure 6E). In this assay, the ssDNA substrate was assimilated into ∼10% of the supercoiled DNA substrate in the absence of caffeine. Addition of caffeine inhibited Rad51 D-loop formation; 50% inhibition was seen at 10 mM and 90% inhibition at 60 mM. Because *in vivo* repair was also inhibited after treatment with 10 mM caffeine but Rad51 was only displaced at a concentration of 30 mM or higher (Figure 1C and D) we hypothesize that caffeine at concentrations lower than 30 mM could inhibit GC by altering D-loop formation without leading to visible loss of the Rad51 filament from the ssDNA *in vivo*, in agreement with previous analysis of mammalian Rad51 (43).

**Figure 7.**
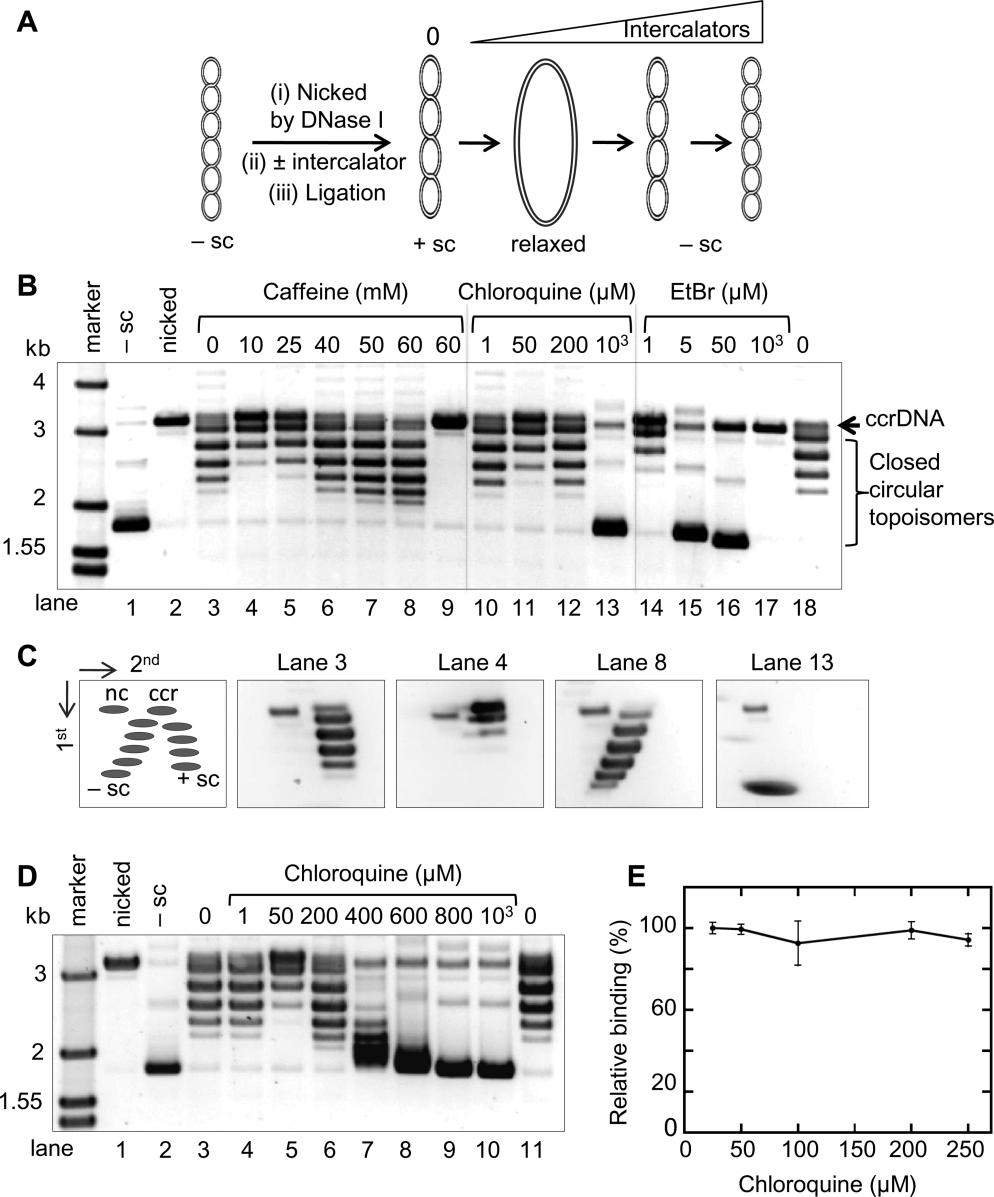
The dsDNA intercalating activity of caffeine does not affect its inhibition of Rad51 binding to ssDNA. (**A**) Diagram representation of an intercalating assay. Negatively supercoiled plasmid was first nicked, then incubate with or without intercalator. The nick was subsequently sealed by ligation to fix any topological changes induced by the intercalator. The resulting topoisomers can be analyzed by agarose gel electrophoresis. Upon binding intercalator, the nicked plasmid unwinds toward the relaxed state, then to the negative supercoiling (–sc) states as the concentration of intercalator increases. (**B**) The effect of caffeine, chloroquine and ethidium bromide on the conformation of dsDNA. Negatively supercoiled plasmid pBlueScript was nicked and religated in the presence of different concentrations of intercalating compounds (refer to diagram in (A)). The reaction products containing topoisomers were separated by electrophoresis on 1% agarose gel. Lanes 1, 2 and 9 have no ligase. ccrDNA: covalently closed, relaxed DNA. (**C**) 2D agarose gel analysis of topoisomers. Schematic diagram of the relative positions of topoisomers resolved on 2D gels is shown; nc, nicked, circular DNA; ccr, covalently closed, relaxed DNA; −sc, negatively supercoiled DNA; +sc, positively supercoiled DNA. Selected samples from reactions in section (B) above (lanes 3, 4, 8 and 13) were analyzed. (**D**) The concentration effect of chloroquine on dsDNA. The assay was carried out as in section (B) above by titrating chloroquine concentration to obtain an intercalating effect similar to that observed for 60 mM caffeine. (**E**) ScRad51 binding to ssDNA assayed by fluorescence polarization in the presence of increasing concentrations of chloroquine. Rad51 was 150 nM, ssDNA was fluorescein-tagged 84 mer (200 nM nucleotides or 2.4 nM). The binding reaction was at 37°C for 30 min. Error bars represent standard deviation from triplicate trials.

Caffeine has been previously shown to intercalate DNA (49). However, Zelensky *et al*. (43) did not find caffeine to intercalate DNA at the concentrations used in their experiments (1–8 mM), which were lower than the concentrations used in our study. We therefore tested the intercalating activity of caffeine at higher concentrations. Using the *in vitro* intercalation assay described by Zelensky *et al*. (43) (Figure 7A) we show that caffeine does intercalate into dsDNA (Figure 7B and C). Caffeine at 10–60 mM changed the starting dsDNA population from positively supercoiled topoisomers to negatively supercoiled topoisomers (Figure 7B and C).

D-loop formation requires two separate activities—the formation of a Rad51 filament on ssDNA and invasion of the Rad51-ssDNA into a homologous dsDNA to create a joint molecule. We first sought to address how intercalation affects Rad51 filament formation. To test this we used chloroquine as a surrogate intercalating agent. By testing different chloroquine concentrations, we found that 200 μM chloroquine has similar dsDNA intercalating activity as 60 mM caffeine (Figure 7B and D). We then used the fluorescence polarization (FP) assay to determine if 200 μM chloroquine can disrupt Rad51-ssDNA association. We found that 200 μM chloroquine did not affect Rad51–ssDNA interaction (Figure 7E).

**Figure 8.**
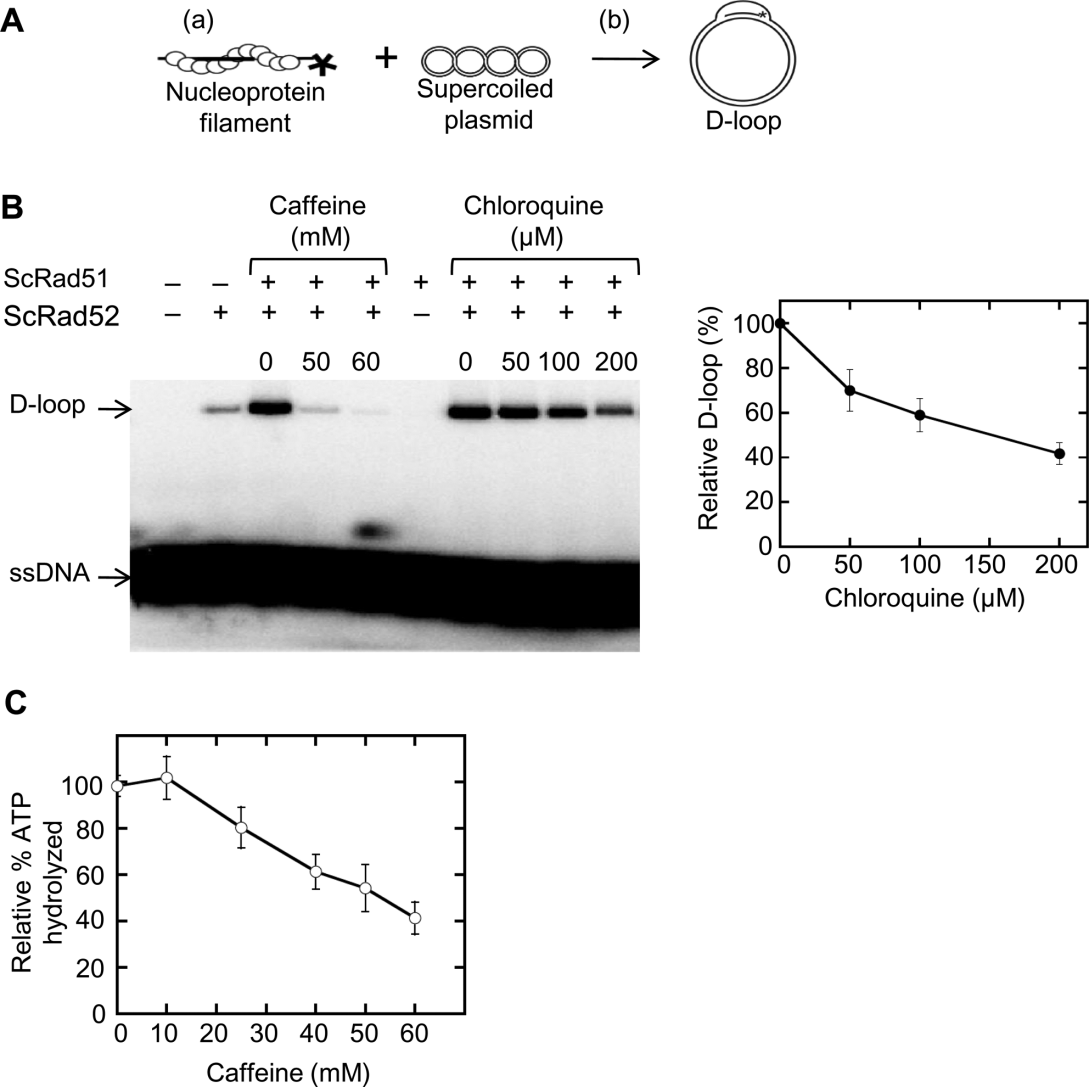
Intercalating partially interfere with D-loop stability. (**A**) A schematic diagram of the D-loop assay. Two partial reactions include (a) Rad51-ssDNA pre-synaptic filament formation and (b) subsequent strand invasion and exchange to form D-loops. (**B**) The D-loop activity of Rad51 (1.2 μM) mediated by Rad52 (2.4 μM) was assayed in the presence of increasing concentrations of caffeine and chloroquine. Autoradiogram of agarose gel separating D-loops from free ssDNA is shown. The relative percent D-loop formed in the presence of chloroquine is plotted. Error bars represent standard deviation from triplicate trials. (**C**) ATP hydrolysis by Rad51 (2.5 μM) in the presence of ssDNA (7 μM nucleotide) with or without caffeine was analyzed. Error bars represent standard deviation from triplicate trials.

Next we tested if D-loop formation is disrupted by intercalation. We used 200 μM chloroquine in the D-loop assay (Figure 8A), 200 μM chloroquine decreased D-loop formation to 40% compared to the control (Figure 8B), whereas 60 mM caffeine decreased D-loop formation to 10% of the untreated control (Figures 6E and 8B). We conclude that intercalation can account for some, but not all, of the effect 60 mM caffeine treatment exerts on D-loops in this biochemical assay. The intercalation activity of caffeine thus does not appear to explain the effect of caffeine on Rad51–ssDNA interaction.

Rad51 has a DNA-dependent ATPase activity that has been shown *in vitro* to be important for dissociation of the Rad51–dsDNA interaction as well as for Rad51-ssDNA filament turnover (17). We therefore tested the effect of 60 mM caffeine on the DNA-dependent Rad51 ATPase activity. We found that 60 mM caffeine decreased Rad51 ATPase activity by ∼60% of untreated samples (Figure 8C). Given that the ATP-bound form of Rad51 has a higher affinity for DNA than the ADP-bound form, these results indicate that the disruptive activity of caffeine cannot be accounted for by stimulation of ATP hydrolysis.

### Overexpression of Rad51 rescues both filament formation and GC

**Figure 9.**
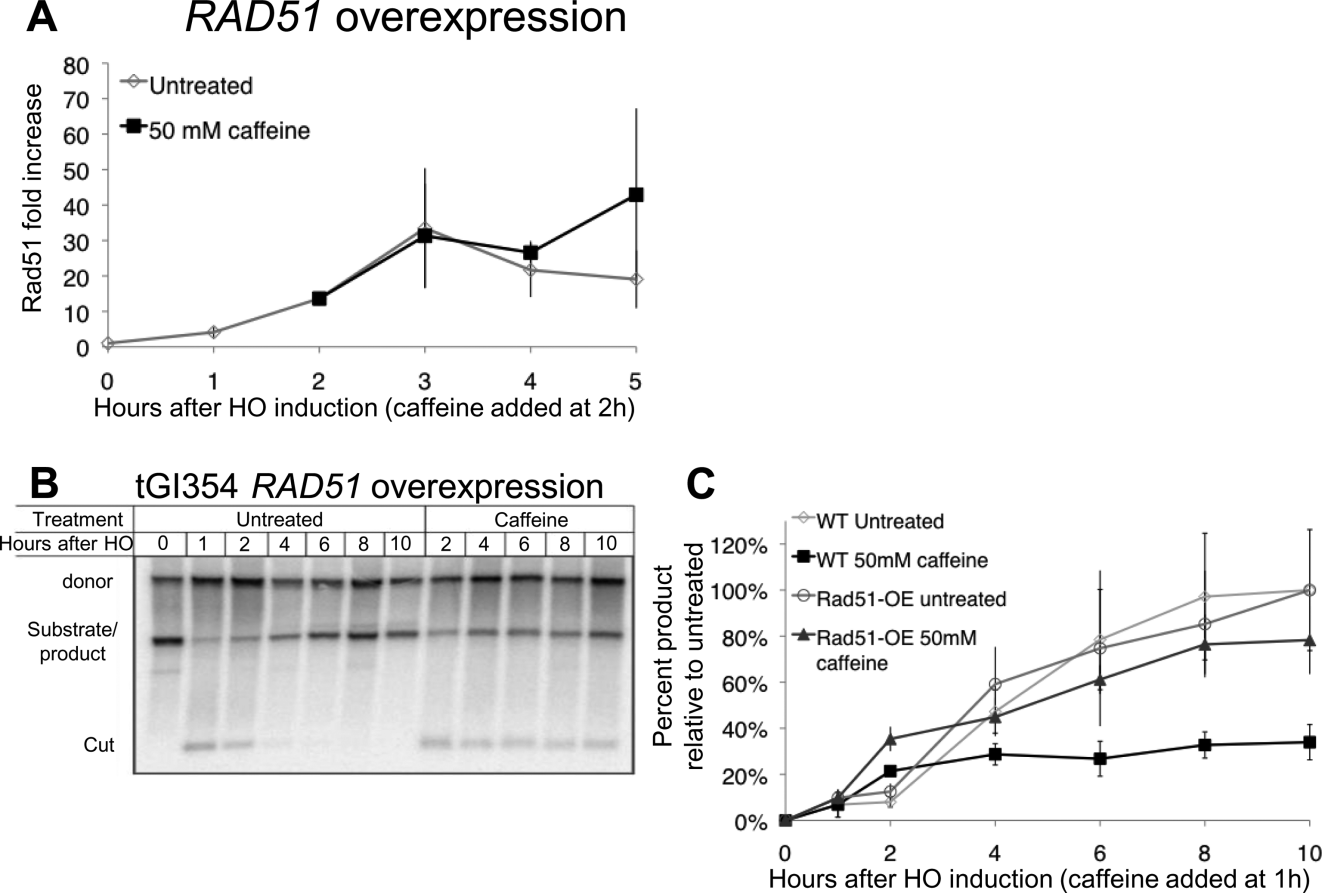
Rad51 overexpression rescues the Rad51 filament and GC. (**A**) Rad51 ChIP 5 kb from a DSB at *MAT* in a donorless strain transformed with a 2μ plasmid containing *RAD51* under the *ADH1* constitutive promoter (pJH657). Error bars represent ranges. (**B**) Repair in tGI354 with *RAD51* overexpression vector. Culture split 1 h after HO induction, half treated with 50 mM caffeine. (**C**) Quantification of repair in tGI354 untreated (diamonds) tGI354 treated with 50 mM caffeine 1 h after HO induction (black squares), tGI354 with *RAD51* overexpression vector (circles) and tGI354 with *RAD51* overexpression vector treated with 50 mM caffeine 1 h after HO induction (triangles). Signal normalized to the maximum signal of the untreated cultures and 0 h. Error bars represent standard deviations.

Although reduced Rad51 levels caused by blocking new protein synthesis do not appear to account for caffeine's eviction of Rad51 from filaments, we asked if increasing Rad51 protein levels could overcome this eviction. We transformed yeast with a 2μ plasmid expressing Rad51 from the constitutive *ADH1* promoter, and assessed Rad51 accumulation around the DSB using ChIP (Figure 9A). Overexpressed Rad51 accumulated on ssDNA following a DSB. The signal reaches a plateau 3 h after HO induction, likely due to saturating the ssDNA. A higher background signal most likely accounts for the observed lower fold increase, as the ratio of IP/non-IP at 0 h is three times higher in RAD51-OE cells than it is in WT (data not shown). Strikingly, caffeine treatment failed to evict Rad51 from the ssDNA when Rad51 was overexpressed. The Rad51 ChIP signal on the ssDNA in the caffeine-treated cells was comparable to that seen in the untreated cells.

The inability of caffeine to evict Rad51 from ssDNA when Rad51 is overexpressed presented us with the ability to test if GC would also be proficient in caffeine-treated cells (Figure 9B and C). We transformed the overexpressing plasmid into a strain that repairs a DSB at *MAT***a** introduced in Chr 5 by using a *MAT***a**-inc donor at the endogenous Chr 3 locus (described in Figure 1A). We induced a DSB 1 h prior to addition of 50 mM caffeine. At this time the DNA close to the DSB is almost fully resected in all the cells (40), and over 90% of the HO recognition sites have been cut (32). Without caffeine, repair in the overexpressing cells was comparable to cells having only a single chromosomal copy of *RAD51*. Following caffeine addition, the cells with only a chromosomal copy of *RAD51* repaired only 30% of the untreated level (Figure 9B and C), and no significant increase in product was seen 1 h after caffeine treatment (2 h post-HO induction) to the end of the experiment. In cells overexpressing Rad51, however, repair was significantly higher and reached 80% compared to the untreated culture (Figure 9C), in spite of the fact that some HO-cut DNA remained unresected. Thus overexpression of Rad51 significantly rescues the GC inhibition caused by caffeine.

### Caffeine treatment abolishes Rad51 foci in mammalian cells

**Figure 10.**
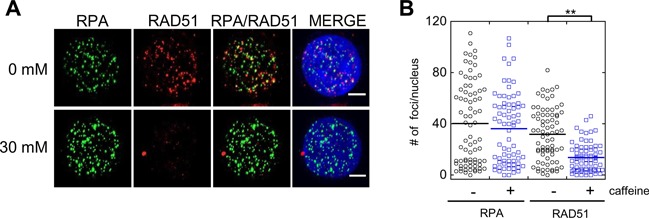
Caffeine treatment evicts Rad51 in HeLa cells. (**A**) Rad51 foci are lost in HeLa cells following caffeine treatment. 30 mM caffeine added to cells 4 h after irradiation (6 Gy). Cells fixed 1 h or after caffeine treatment and immuno-stained with α-Rad51 or α-RPA antibodies. (**B**) Quantification of Rad51 and RPA foci. **P value < 0.005 Wilcoxon Rank Sum test.

In an accompanying paper (50) we showed that caffeine impairs resection in mammalian cells. Here we asked if caffeine treatment results in eviction of RAD51 from ssDNA in HeLa cells, by determining if caffeine treatment would lead to loss of Rad51 foci already assembled on damaged DNA. HeLa cells were treated with 30 mM caffeine 4 h after irradiation. We then fixed and double stained cells for RPA and Rad51 1 h after caffeine treatment. When HeLa cells were treated with 30 mM caffeine the number of Rad51 foci significantly decreased from an average of 32 ± 3 RAD51 foci per nucleus to 11 ± 1 foci per nucleus. In contrast to the effect of caffeine on RAD51 foci, there was no significant difference in the number of RPA foci between caffeine treated (36 ± 1 foci/nucleus) and untreated (40 ± 5 foci/nucleus) cells (Figure 10A and B). This result suggests that the effects of caffeine on Rad51 protein localization are similar in yeast and mammals. The results further raise the possibility that RAD51 filament disruption is a conserved mechanism by which caffeine inhibits HR.

## DISCUSSION

Caffeine treatment was found by us and others to inhibit HR by mechanisms distinct from the drug's established ATM and ATR inhibition (36–38,43). In an accompanying paper we show that caffeine also impairs resection, which precedes Rad51 loading, by causing the rapid loss of the Sae2 and Dna2 resection proteins thus inhibiting repair by SSA. Here we show that caffeine inhibits GC, by eviction of the Rad51 filament at higher levels of caffeine. The results are also consistent with reduced filament function at lower levels of caffeine, as was previously proposed from experiments in mammalian cells (43).

The eviction of Rad51 following caffeine treatment is rapid, taking place in an hour or less. Rad51 is evicted independently of the inhibition of Mec1 and Tel1. Caffeine's effect cannot be explained by interfering with either of the known pathways that regulate Rad51 filament assembly on ssDNA, the filament dissociating activity of Srs2 or the removal of Rad51 non-specifically bound on dsDNA, by Rad54, Rdh54 or Uls1. Rad51's eviction also did not depend on its ATPase activity or having an intact site II domain to bind dsDNA. Moreover, caffeine did not impair the ability of RPA or Rad52 to associate with ssDNA, indicating that sufficient ssDNA forms to serve as a substrate for protein binding. Indeed, caffeine treatment resulted in improved association of Rad52 with the ssDNA close to the DSB. The increased ChIP signal of Rad52 may indicate that without the ability to replace RPA with Rad51 filament, Rad52 remains bound to RPA. Rad52 does not apparently promote Rad51 eviction under caffeine treatment, as eviction occurred even when Rad52-AID was abolished by addition of auxin.

Rad51 expression is upregulated in response to a single DSB, and this upregulation is required for continuous loading of Rad51 on the ssDNA. This expression of Rad51 following a DSB is controlled by the DNA damage checkpoint. The lower level of Rad51 signal around an irreparable DSB in the *mec1*Δ *tel1*Δ *sml1*Δ strain may reflect lower Rad51 protein pools available to establish a filament when there is no damage-induced increase in Rad51 expression, and these lower levels of Rad51 may contribute to the lower efficiency of GC in the *mec1*Δ *tel1*Δ *sml1*Δ strain. However there are likely to be other contributing factors, including the faster resection in a *mec1*Δ *tel1*Δ *sml1*Δ strain (50), that may lead to more dispersed Rad51 loading over a larger ssDNA substrate. Furthermore, the absence of Mec1 or Tel1-dependent modifications of several recombination proteins cannot be excluded (82,83). Like CHX, caffeine treatment prevented the upregulation of Rad51 total protein pools following a DSB. However, the complete eviction of Rad51 from the ssDNA as seen in the ChIP data differs from the signal seen after CHX treatment. Moreover, caffeine treatment 4 h after HO induction did not result in loss of protein levels but did lead to Rad51 eviction. We conclude that inhibition of protein synthesis may contribute to inhibition of GC after caffeine treatment, but alone is not sufficient to cause eviction of Rad51 or explain the more complete inhibition of GC following caffeine treatment.

Although overexpression of Rad51 enables Rad51 to maintain a filament on ssDNA in the presence of caffeine and partially rescues the repair defect, it is unlikely that caffeine exerts its effect on Rad51 primarily by altering protein levels. Levels of Rad51 after caffeine treatment did not decrease below the basal levels and were similar to the levels seen with CHX, which allow the retention of a stable Rad51 filament. Thus, while caffeine treatment prevents the induction of elevated levels of Rad51 protein synthesis, its eviction must be explained by another mechanism. The fact that Rad51 overexpression can override the presence of caffeine suggests that when Rad51 is present at abnormally high concentrations the unbound Rad51 can replace the Rad51 molecules evicted from the ssDNA by caffeine. This hypothesis is consistent with the possibility that caffeine can directly inhibit Rad51–ssDNA interaction.

Caffeine was found to inhibit both Rad51 DNA binding and D-loop activity in biochemical assays, supporting the conclusion that direct inhibition of Rad51–ssDNA interaction makes a significant contribution to the *in vivo* effects of caffeine on GC. A previous study of caffeine-mediated inhibition of human RAD51 (HsRAD51) (43) did not detect direct disruption of the ssDNA-bound HsRad51 using an electrophoretic mobility shift assay (EMSA) at concentrations of up to 10 mM, but showed that 4 mM caffeine blocked D-loop formation by altering the activity of HsRAD51 filaments such that they had an increased tendency to form stable ternary complexes with heterologous duplex DNA. Using FP we observed dissociation of yeast Rad51 (ScRad51) from ssDNA. At 25 mM 15–20% of the ssDNA-bound ScRad51 is lost. We observed more efficient inhibition of ScRad51 D-loop activity at 25 mM (80%) than its DNA binding activity. At 10 mM caffeine only 4% of the ssDNA-bound ScRad51 signal was lost, but ∼50% of ScRad51-mediated D-loops were lost. Thus, the mechanism through which caffeine inhibits Rad51 function may be complex; lower concentrations of caffeine may block D-loop formation by altering the normal activity of ssDNA-bound RAD51 in both human and yeast, while higher concentrations of caffeine might block D-loop formation by promoting dissociation of Rad51 filaments. Alternatively, it is possible that at lower caffeine concentrations ssDNA-bound Rad51 could be destabilized sufficiently to interfere with the fidelity of homology search and D-loop formation but not sufficiently to be detected as loss of signal by either ChIP or EMSA (and detected as weak loss by FP), whereas at higher caffeine concentrations the destabilization of ssDNA-bound Rad51 could be detected as loss of signal by these techniques. We note that the degree of inhibition of D-loop formation that we observe for ScRad51 is somewhat less than that reported for HsRAD51, which may reflect the fact that the assay for ScRad51 requires Rad52 and/or the fact that the ScRad51 assay does not include non-physiologically high levels of Ca^2+^ which are required for HsRAD51 D-loop activity. Taken together, our data strongly suggest that caffeine acts directly on Rad51 to inhibit GC.

Previously Zelenskey *et al*. reported that caffeine at 1–8 mM does not intercalate dsDNA. We show that at the concentrations that we use in this study caffeine did intercalate dsDNA, albeit more weakly than chloroquine or ethidium bromide. Chloroquine failed to dismantle Rad51–ssDNA interaction but did, to some extent, interfere with D-loop formation. This shows that at least some of the D-loop disruption by caffeine can be attributed to intercalation of dsDNA, however the disruption of the Rad51–ssDNA interaction is independent of intercalation. Moreover we show that caffeine reduces Rad51 ATPase activity. This could be a result of disrupting the Rad51–DNA interaction, as Rad51's ATPase activity is DNA dependent, or it could be a consequence of interfering directly with Rad51-ATP binding which is required for filament formation. In this model, Rad51 forms a filament and then dissociates by hydrolyzing ATP. In the presence of caffeine Rad51 binds caffeine or a metabolite of caffeine instead of ATP and cannot return to the filament, leading to the eventual dissolution of the entire filament.

The effects of caffeine that we have described in budding yeast appear to be evolutionarily conserved. When caffeine was added to HeLa cells prior to induction of DSBs, it blocked both Rad51 and RPA foci formation, consistent with a defect in resection, as we describe for yeast in an accompanying paper. Here we show that, if caffeine is added several hours after DSB induction, Rad51 foci were markedly diminished, but without a concomitant loss of RPA foci, analogous to the ChIP results presented here for budding yeast Rad51. Moreover, Rad51 eviction in yeast and mammals takes place at similar concentrations of caffeine.

Caffeine was previously shown to sensitize p53-deficient cancer cells to ionizing irradiation. The data presented here further our mechanistic understanding of caffeine-mediated radiosensitivity, informing the efforts to develop small molecules that target HR as a treatment for cancer (84–88).

## SUPPLEMENTARY DATA

Supplementary Data are available at NAR Online.

SUPPLEMENTARY DATA
