# Supplementary material for: Caffeine inhibits gene conversion by displacing Rad51 from ssDNA
Source: Nucleic Acids Res. 2015 May 27;43(14):6902–18. doi: 10.1093/nar/gkv525 (PMC4538809; doi:10.1093/nar/gkv525)
Supplement: SUPPLEMENTARY DATA [file supp_43_14_6902__index.html]

Caffeine inhibits gene conversion by displacing Rad51 from ssDNA — Caffeine inhibits gene conversion by displacing Rad51 from ssDNA — Caffeine inhibits gene conversion by displacing Rad51 from ssDNA — SUPPLEMENTARY DATA 

# Caffeine inhibits gene conversion by displacing Rad51 from ssDNA

## SUPPLEMENTARY DATA

- SUPPLEMENTARY DATA
