# Supplementary material for: Caffeine inhibits gene conversion by displacing Rad51 from ssDNA
Source: Nucleic Acids Res. 2015 May 27;43(14):6902–18. doi: 10.1093/nar/gkv525 (PMC4538809; doi:10.1093/nar/gkv525)
Supplement: SUPPLEMENTARY DATA [file supp_gkv525_nar-03490-d-2014-File012.pdf]

Supporting information

Figure S1

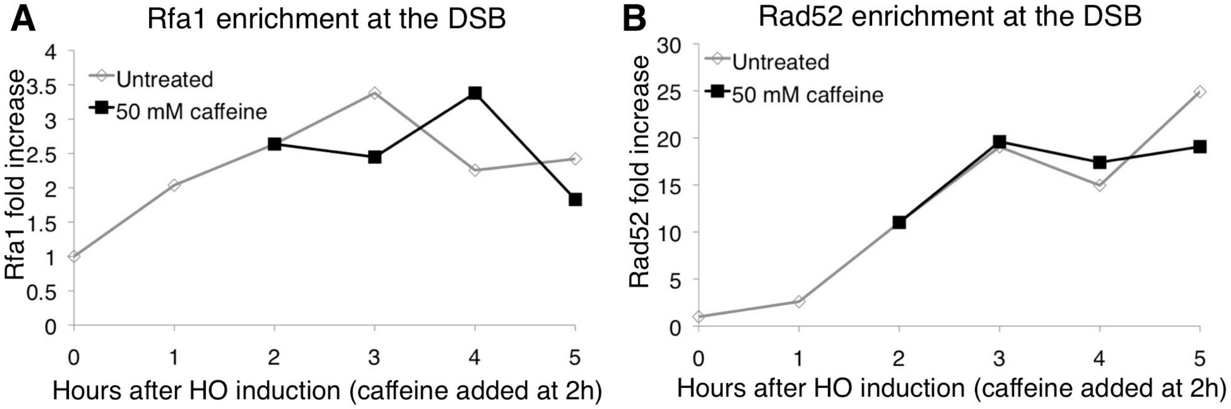

Supplemental Figure 1 – Caffeine treatment does not lead to loss of Rfa1 or Rad52 from ssDNA

A) Rfa1 ChIP at Chr 6 DSB (irreparable) in strain YML002. ChIP signal measured 5 kb proximal to the DSB.

B) Rad52 ChIP 5 kb proximal to the DSB at *MAT* in JKM179. ChIP signal measured 5 kb proximal to the DSB at *MAT.*

Figure S2

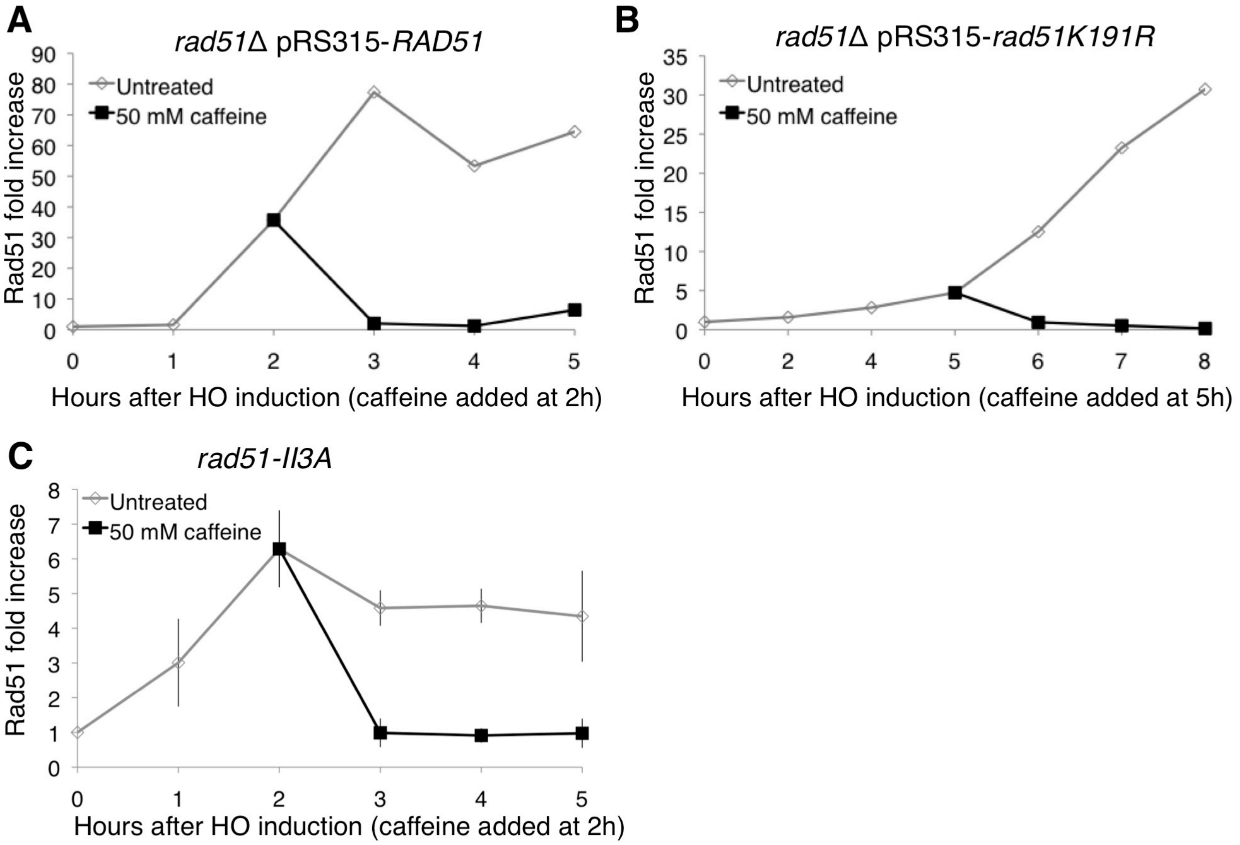

Supplemental Figure 2

A) Rad51 loading on ssDNA. Rad51 is expressed from a single copy centromere containing plasmid. WT RAD51 is deleted. ChIP signals measured 5 kb from the DSB.

B) rad51-K191R loading on ssDNA. rad51-K191R is expressed from a single copy centromere containing plasmid. WT RAD51 is deleted. ChIP signals measured 5 kb from the DSB.

C) Rad51 ChIP 5 kb from a DSB at *MAT* in a donorless *rad51-II3A* strain. 50 mM caffeine added 2 h after HO induction. Error bars represent ranges.

Figure S3

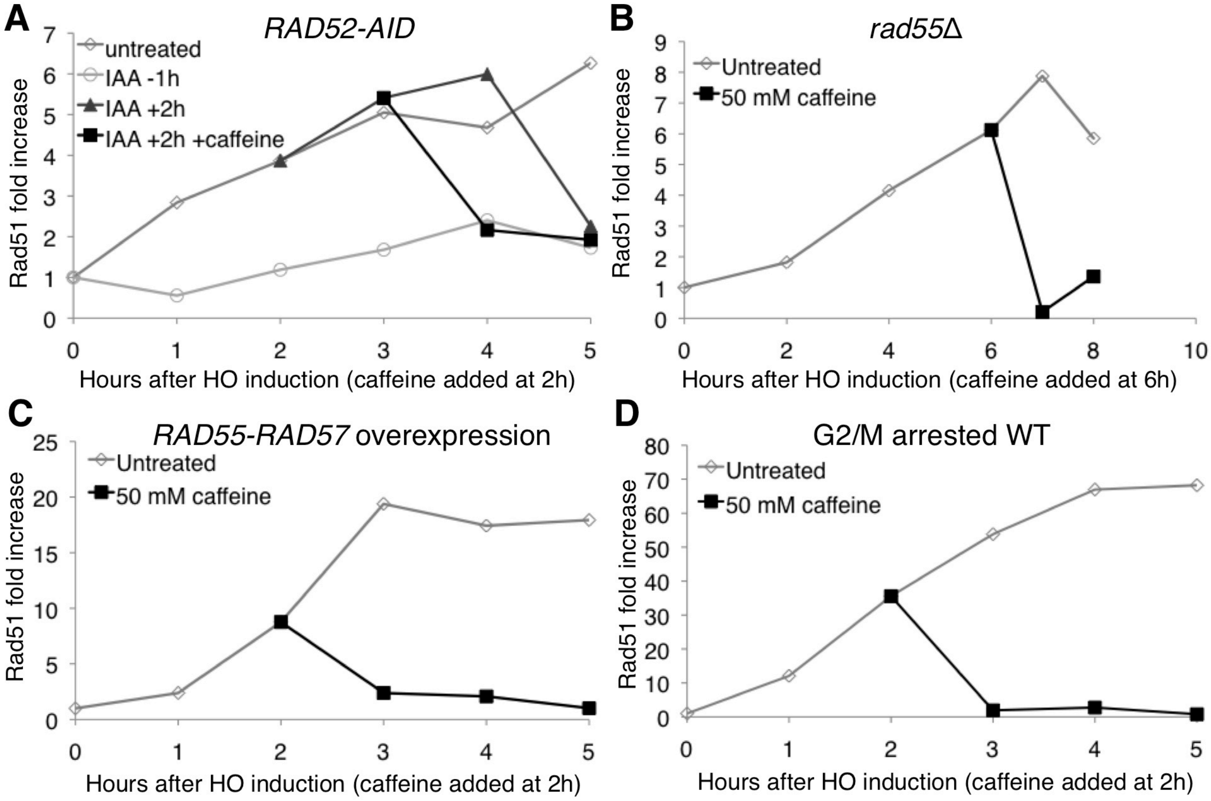

Supplemental Figure 3 - Caffeine treatment leads to Rad51 eviction independently of factors that facilitate Rad51 filament formation.

A) Rad51 loading 5 kb from the DSB in a strain containing Rad52 with an auxin inducible degron (AID). 500 µM indole-3-acetic acid (IAA) added to the media 1 h before HO induction (blank diamonds) or 2 h after HO induction (triangles). 50 mM caffeine added 1 h after IAA treatment (3 h after HO induction).

B) Rad51 loading in 5 kb from the DSB in a *rad55*Δ strain. 50 mM caffeine were added 6 h after HO induction.

C) Rad51 loading 5 kb from the DSB in a in a strain containing Rad55-Rad57 overexpressing plasmid. 50 mM caffeine were added 2 h after HO induction.

D) Caffeine evicts Rad51 in cells arrested in nocodazole. 15 µg/ml nocodazole added to the media 3 h prior to HO induction (95% dumbbells). 50 mM caffeine added 2 h after HO induction.

Figure S4

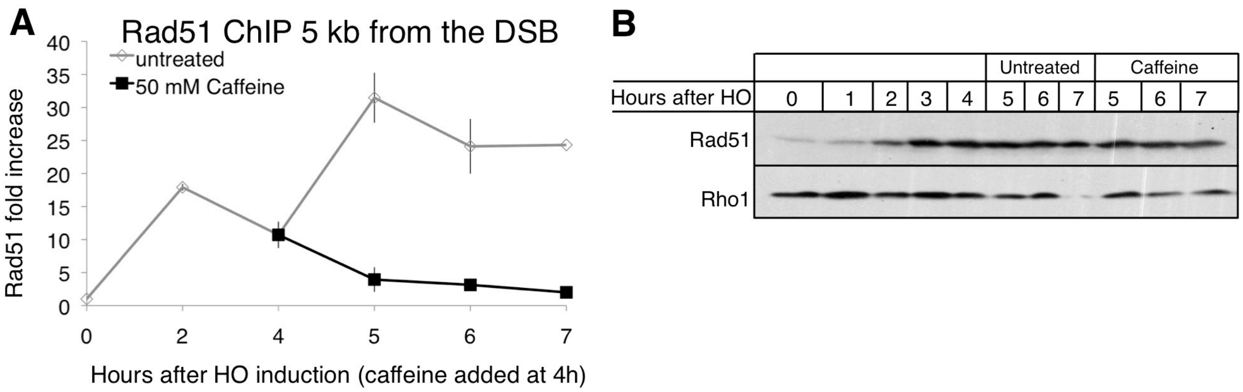

Supplemental Figure 4 – Caffeine evicts Rad51 without lowering Rad51 protein levels

A) Rad51 loading 5 kb from the DSB in a in a donorless strain. 50 mM caffeine were added 4 h after HO induction. Error bars represent ranges.

B) Western blot for Rad51. 50 mM caffeine were added 4 h after HO induction.

Table I – Strain list

Strain name	Genotype
JKM179	*hoΔ hml::ADE1 MATα hmr::ADE1 ade1-110 leu2,3-112 lys5 trp1::hisG ura3-52 ade3::GAL:HO*
JKM139	*JKM179 isogenic, MATa*
YML002	*JKM139 (HO cut site deleted) Cen3HOcs::HPH 2 kb homology to the left of the HOcs inserted to the right of Cen3, 97700-97800 Ch6::HOcs-NAT*
tGI354	*JKM139 MATa-inc (+CA), arg5,6::MATa-HPH*
MT03	*hoΔ HMLα MATα HMRα-BamHI::URA3 ade1 leu2 trp1::hisG ura3-52 ade3::GAL:HO*
YJL112	*MT03 tel1::TRP1 mec1::NAT sml1::KAN*
YFD0918	*JKM179 atg1::KAN*
YFD0247	*JKM139 srs2::LEU2*
AWY313	*JKM179 sgs1::KAN*
YSL305	*JKM139 rad55::LEU2*
MT101	*JKM139 ura3-52::TIR-LEU2 rad52-AID::KAN*
MT104	*JKM179 RAD52-FLAG::KAN*
MT109	*JKM179 pADH1-RAD51 LEU2 2 µ*
MT113	*JKM179 pRAD55-RAD57 URA3 2 µ*
MT121	*tGI354 pADH1-RAD51*
MT123	*JKM179 rad51::HPH pRAD51-LEU2*
MT124	*JKM179 rad51::HPH prad51K191R-LEU2*
MT127	*hoΔ HMLα MATa hmr::ADE1 ade1-110 leu2,3-112 lys5 trp1::hisG ura3-52 ade3::GAL:HO rad54::KAN rdh54::URA3 uls1::LEU2*
MT151	*JKM179 RAD51-II3A::TRP1*
